# The impacts of social determinants of health and cardiometabolic factors on cognitive and functional aging in Colombian underserved populations

**DOI:** 10.1007/s11357-023-00755-z

**Published:** 2023-02-28

**Authors:** Hernando Santamaria-Garcia, Sebastian Moguilner, Odir Antonio Rodriguez-Villagra, Felipe Botero-Rodriguez, Stefanie Danielle Pina-Escudero, Gary O’Donovan, Cecilia Albala, Diana Matallana, Michael Schulte, Andrea Slachevsky, Jennifer S. Yokoyama, Katherine Possin, Lishomwa C. Ndhlovu, Tala Al-Rousan, Michael J. Corley, Kenneth S. Kosik, Graciela Muniz-Terrera, J. Jaime Miranda, Agustin Ibanez

**Affiliations:** 1 Global Brain Health Institute (GBHI), University of California San Francisco (UCSF), San Francisco, CA USA; 2 Pontificia Universidad Javeriana (Ph.D. Program in Neuroscience, Department of Psychiatry), Bogotá, Colombia; 3 Center of Memory and Cognition Intellectus, Hospital Universitario San Ignacio, Bogotá, Colombia; 4 Latin American Brain Health Institute (BrainLat), Universidad Adolfo Ibañez, Santiago de Chile, Chile; 5 Cognitive Neuroscience Center (CNC), Universidad de San Andrés, and CONICET, Buenos Aires, Argentina; 6 Department of Neurology, Massachusetts General Hospital and Harvard Medical School, Boston, MA USA; 7 Institute for Psychological Research, University of Costa Rica, Sabanilla, Costa Rica; 8 Department of Neurology, Memory and Aging Center, Weill Institute for Neurosciences, University of California, San Francisco, CA USA; 9 Facultad de Medicina, Universidad de los Andes, Bogotá, Colombia; 10 Instituto de Nutrición Y Tecnología de los Alimentos, Universidad de Chile, Avenida El Líbano 5524, Macul, Santiago, Chile; 11 Mental Health Department, Hospital Universitario Fundación Santa Fe de Bogotá, Memory Clinic, Bogotá, Colombia; 12 Neuropsychology and Clinical Neuroscience Laboratory (LANNEC), Physiopathology Department - Institute of Biomedical Sciences (ICBM), Neurocience and East Neuroscience Departments, Faculty of Medicine, University of Chile, Santiago de Chile, Chile; 13 Geroscience Center for Brain Health and Metabolism, (GERO), Santiago de Chile, Chile; 14 Memory and Neuropsychiatric Center (CMYN), Memory Unit - Neurology Department, Hospital del Salvador and Faculty of Medicine, University of Chile, Santiago de Chile, Chile; 15 Servicio de Neurología, Departamento de Medicina, Clínica Alemana-Universidad del Desarrollo, Santiago de Chile, Chile; 16 Department of Medicine, Division of Infectious Diseases, Weill Cornell Medicine, New York, NY USA; 17 Feil Family Brain and Mind Research Institute, Weill Cornell Medicine, New York, NY USA; 18 Herbert Wertheim School of Public Health, University of California San Diego, La Jolla, CA USA; 19 Neuroscience Research Institute. Department of Molecular Cellular and Developmental Biology, University of California Santa Barbara, Santa Barbara, CA USA; 20 Edinburgh Dementia Prevention, University of Edinburgh, Edinburgh, UK; 21 Department of Primary Care, Ohio University, Athens, USA; 22 CRONICAS Center of Excellence in Chronic Diseases, Universidad Peruana Cayetano Heredia, Lima, Peru; 23 Department of Medicine, School of Medicine, Universidad Peruana Cayetano Heredia, Lima, Peru; 24 Faculty of Epidemiology and Population Health, London School of Hygiene and Tropical Medicine, London, UK; 25 The George Institute for Global Health, UNSW, Sydney, Australia; 26 Trinity College Dublin (TCD), Dublin, Ireland

**Keywords:** Social determinants of Health, Cardiometabolic factors, Cognition, Functionality, National Aging Population Survey

## Abstract

Global initiatives call for further understanding of the impact of inequity on aging across underserved populations. Previous research in low- and middle-income countries (LMICs) presents limitations in assessing combined sources of inequity and outcomes (i.e., cognition and functionality). In this study, we assessed how social determinants of health (SDH), cardiometabolic factors (CMFs), and other medical/social factors predict cognition and functionality in an aging Colombian population. We ran a cross-sectional study that combined theory- (structural equation models) and data-driven (machine learning) approaches in a population-based study (*N* = 23,694; *M* = 69.8 years) to assess the best predictors of cognition and functionality. We found that a combination of SDH and CMF accurately predicted cognition and functionality, although SDH was the stronger predictor. Cognition was predicted with the highest accuracy by SDH, followed by demographics, CMF, and other factors. A combination of SDH, age, CMF, and additional physical/psychological factors were the best predictors of functional status. Results highlight the role of inequity in predicting brain health and advancing solutions to reduce the cognitive and functional decline in LMICs.

**Supplementary Information:**

The online version contains supplementary material available at 10.1007/s11357-023-00755-z.

## Introduction

Recent global initiatives call for further research on determinants that may be detrimental to brain health in aging populations, especially in low- and middle-income countries (LMICs) [1]. By 2050, the proportion of people living with dementia will increase by around 75% in western Europe and by around 200% in Colombia and other countries in Latin America [2]. Although two thirds of individuals with dementia live in LMICs, few studies have systematically assessed these factors in those countries [2, 3]. The assessment of socioeconomic, physical, and mental health determinants on cognitive and functional capacity in aging populations in LMICs is critical to better understand the role of inequity on brain aging and to support mitigation strategies [4].

Brain health and aging are shaped by social determinants of health (SDH)—physical, medical, and nutritional factors associated with inequity [5]. Negative SDH factors that could increase brain health risks include poor social and economic resources (9), reduced social participation factors [6], limited social access [7], and exposure to social adversities [8, 9]. Brain aging inequities are also impacted by cardiometabolic risk factors (CMF) such as cardiovascular diseases, obesity, and diabetes [10, 11]. The increased cardiometabolic risk in LMICs (in comparison with high-income countries, HICs) has been associated with increased dementia rates [10, 12–14]. Many risk factors related to SDH and CMF have been shown to account for 40% of dementia prevalence in HICs [12], but this percentage increases to 56% across LMICs [14]. Thus, SDH and CMF are critical for characterizing aging in terms of cognitive and functional levels across LMICs.

Previous research on health disparities conducted in LMICs, however, presents several caveats. Most studies in LMICs do not investigate combined and exhaustive sources of inequity (i.e., multiple SDH and CMF) in predicting both cognition and functionality in normal aging. Studies of LMICs have only reported independent associations between SDH and cognition [15] and between CMF and cognition [10, 16], precluding their joint assessment which is needed to understand the interplay of social and individual-level medical factors. Additionally, most of these studies have only assessed patients with dementia, underestimating the study of normal aging. Although some studies have found associations between chronic conditions and functional status in LMICs [17, 18], no such study in LMICs has systematically assessed the simultaneous impact of different inequity signatures on functional capacity. Importantly, to the best of our knowledge, no population-based study has concurrently evaluated combined sources of inequity (and their potential interactions) to determine brain health outcomes in terms of cognition and functionality in LMIC settings.

**Fig. 1:**
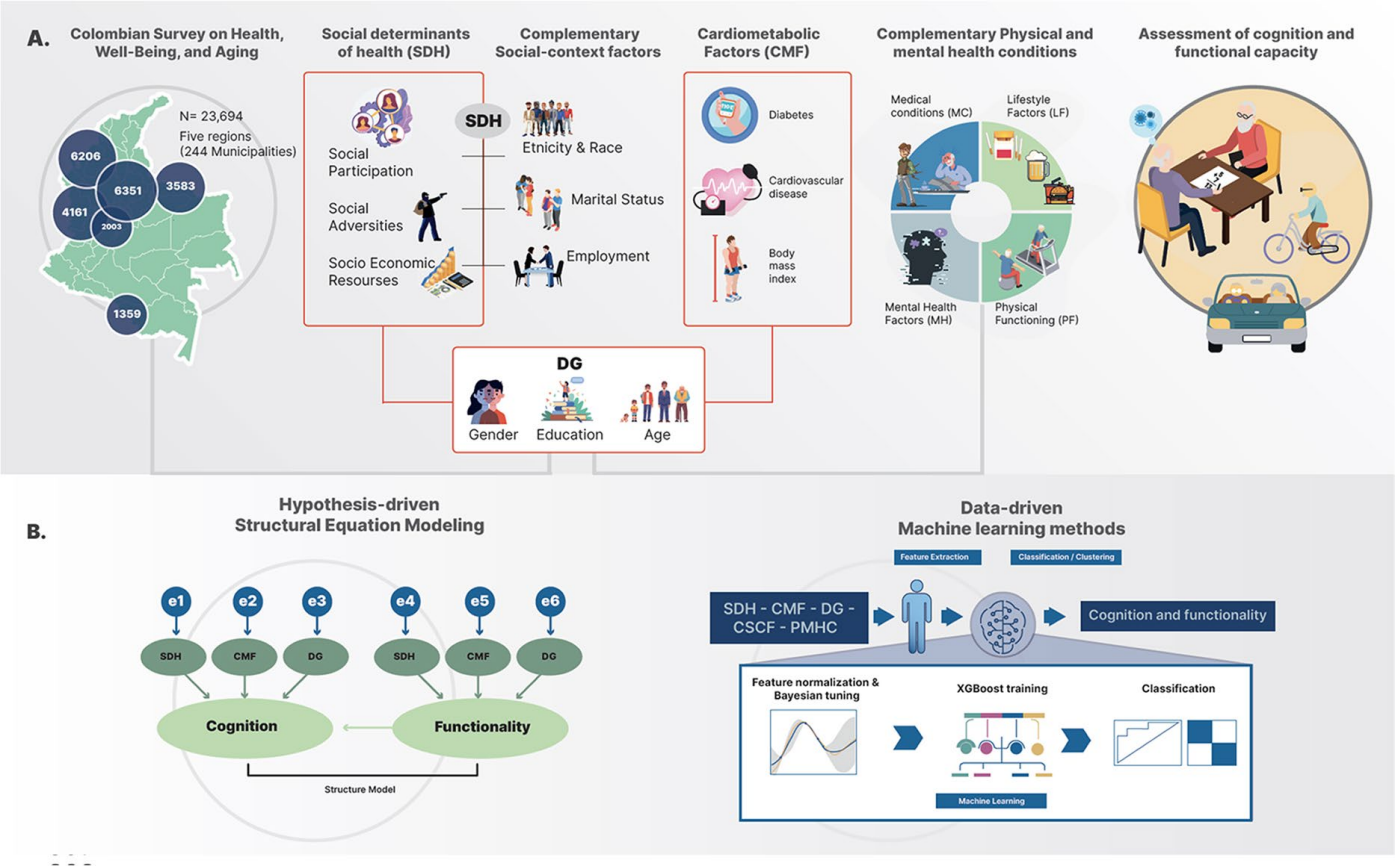
Study design, methodology, and analyses. **A** The general structure of the Colombian Survey on Health, Well-Being, and Aging (SHWA). The top left panel depicts the Colombian regions (Central, Pacific, Amazonian, Atlantic, and Bogota regions) where data were collected to complete 23,694 individuals. The middle panel in **A** shows the different types of factors assessed in the study. The predictors included in the theory-driven approach (DG, SDH, and CMF) are detained in the red boxes. Moreover, additional health variables factors were assessed in the data-driven approach (blue boxes). The top right panel in **A** shows the two outcomes assessed in this study (cognitive and functionality levels). **B** A simplified model of the theory-driven approach using the multigroup structural equation modelling procedure. **C** The machine learning procedures used in the data-driven analyses. These analyses involve the implementation of a XGBoost procedure and progressive feature elimination procedure. DG, socio-demographic conditions; SDH, social determinants of health; CMF, cardiometabolic factors; MH, mental health factors; PF, physical functioning; LF, lifestyle factors

The current study aimed to bridge these gaps by assessing the impact of inequity signatures (indexed by demographics, SDH, and CMF) and how they interact to predict brain health outcomes in an underserved LMIC-Colombia (Fig. 1). In particular, we assessed two relevant markers of brain health, cognitive performance and functional capacity (involving different capacities including instrumental activities skills and functional mobility [19, 20]).

Colombia could be considered a suitable LMIC for the present research question. First, it exhibits a high level of social inequality. Data on the degree of inequality in wealth distribution as measured by the Gini coefficient reached 50.4, ranking Colombia in the 15th place across the world in 2015 by the World Bank) [21]. Second, Colombia exhibits high rates of social adversities related to extreme violence and forced displacement [22]. Additionally, the country also presents a high burden of chronic non-communicable diseases and CMF [23].

We analyzed a sample of 23,694 individuals (mean age = 69.8 years, SD = 7.9 years) collected from different regions (Central, Pacific, Amazonian, Atlantic, and Bogotá) in the 2015 Colombian National Survey on Health, Well-Being, and Aging (in Spanish SABE: Salud, Bienestar & Envejecimiento, 2015) [24]. We combined two different approaches, including theory-driven (multigroup-structural equation modeling) and data-driven (machine learning) methods, to accurately identify the determinants of cognition and functionality.

First, theory-driven methods aimed to assess the extent to which a conceptual set of direct and conceptually based measures (latent predictors based on multiple measurable SDH and CMF) can trace complex interactions and predict cognition and functional status. Structural equation modeling allows for detection of causal associations between different variables, including direct measures and grouped variables (also named latent variables) and outcomes. It operates as a regression model that examines causal relationships among multiple variables while controlling the measurement error [25, 26]. Second, data-driven approaches (machine learning procedures based on an XGBoost classifier [27]) were employed to assess the weight of each factor in predicting cognition and functional status. Machine learning helped test the significance of multiple variables associated with brain health outcomes. Machine learning methods treat all factors equally; each is assigned the same weight as a potential predictor. This avoids the stratification of factors based on a priori theoretical hierarchies [28–30]. Moreover, machine learning approaches adequately assess data multidimensionally and identify interactions between potential predictors of an outcome [28–30]. Previous studies suggested complex interactions between demographics, SDH, lifestyle habits, and medical outcomes. Relatedly, increased medical risks and poor lifestyle habits are observed in individuals with fewer social resources and negative SDH [25, 31]. Considering this scenario, we complemented theory-driven approaches in our study by including machine learning procedures that help to address complex interactions between factors and brain health outcomes.

Previous studies have assessed potential predictors of brain health outcomes in Colombia and other LMICs (see Supplementary Information S1 and Table S1 for a review of studies evaluating predictors of cognition and functionality). Most of those studies involve national surveys of aging [32–35]. In terms of outcomes, cognition is usually reported with the MMSE [32, 33, 36–39] (although a few used combined tasks [34, 40]). Regarding functionality, most studies included scales assessing daily life activities, such as the Lawton and Brody scale [41–43]. Considering the predictors, a large set of studies has focused on risk factors, including motor [38] and muscular functioning [32], education [41] or medical conditions [18]. Beyond these large sets of studies of aging in Colombia and LMIC, multiple questions remain unanswered.

According to the Lancet Commission for the Dementia prevention, intervention, and care: 2020 report [12], the top critical potential factors associated with brain health include demographic factors (sex, age, education), lifestyle factors (smoking, alcohol consumption), medical conditions (hypertension, diabetes, obesity, perception skills, mental health), and SDH (income, isolation, health access) [12, 44, 45]. Most studies assessing predictors of cognition and functionality, including those studies using 10/66 survey [44, 46], have found different predictor rankings across countries. Moreover, those studies did not combine analyses assessing different brain health outcomes. Five dementia risk factors were more prevalent in these LMICs than in worldwide estimates: less childhood education, smoking, hypertension, obesity, and diabetes [44–47].

In Colombia, previous studies have reported this SABE survey [22, 33, 39, 48] and considered cognition (measured with MMSE [18, 33, 39, 49]) and functionality (measured with Lawton and Brody and Barthel [50–52]). However, those studies assessed the relation between one single risk factor and cognition or functionality [18, 32, 33, 38, 52]. Also, they found independent associations between CMF and cognition and functionality [18], SDH, demographics, and cognition [39] and associations between motor [38] and muscle functioning [32] and cognition [33]. To the best of our knowledge, no single study has simultaneously investigated the associations between multiple potential determinants and cognition and functionality in Colombia. Our study adds to previous evidence a systematic analysis of multiple combined factors potentially associated with cognition and functionality using a novel combination of theory- and data-driven approaches. Moreover, our study contributes with different methodological approaches to understand the how various related factors interact with each other to predict cognition and functionality, avoiding analyses of associations between isolated factors and outcomes.

We hypothesized that aside from classical demographic factors (i.e., age, sex, and education level), the SDH and CMF would successfully predict cognition and functional status. We expected partially convergent results between theory- and data-driven approaches. Theory-driven analyses may show significant complex interactions between integrated conceptual constructs of demographics, SDH, and CMF in predicting cognition and functional capacity. Moreover, considering the increased social inequalities in the population being studied, we hypothesized that SDH would have a more considerable weight in determining cognition and functional capacity than CMF and other related physical/mental health factors. We anticipated that those effects should be consistently revealed by both theory- and data-driven analyses.

## Methods

### Study design

Cross-sectional national study.

### Setting

We conducted a data analysis of a cross-sectional database from the Colombian Survey on Health, Well-Being, and Aging [24]. This is the first population-based cross-sectional national study on community-dwelling older adults’ health, aging, and well-being. Participants were selected following a probabilistic, clustered, stratified, and multistage design. All interviews were performed face-to-face, between April and September 2015.

### Participants

The sample included four selection stages: (1) the identification of municipalities as primary sampling units, (2) randomized selection of area segments (i.e., blocks) within primary sampling units, (3) choice of housing units within the secondary sampling units, and (4) randomized selection of the household units from the list taken in the third procedure. This survey followed the general framework of the Colombian national surveys system of the Ministry of science of Colombia. The initial estimated sample size was 24,553 individuals, assuming an 80% of responses from a target sample (*N* = 30,691 individuals). Response proportion ranged from 62% in urban areas to 77% in rural sites. All interviews were performed face to face. The final sample size, including 244 rural and urban municipalities across all departments (i.e., states) of the country, comprised 23.694 elderly Colombians representative of the total population. Participants were included if they were 60 + years of age, were capable of communicating with the research team, and provided written informed consent. Individuals with low scores in the MMSE (below 13 points) were excluded at the beginning of the interview and followed a proxy interview (supported by caregivers), as those scores were considered indicative of inability to complete the study procedures. The percentage of interviews applied to proxies was 17.5%. In this study, we only included individuals able to complete the total number of screening questions.

This study was approved (code 2013–764) by the Colombian ministry of science and the Ministry of Health and Social Protection. The Institutional Review Boards of *Universidad de los Andes*, Bogotá, Colombia (code ID 1114/2019) and *Universidad Javeriana*, Bogotá, Colombia (code FM773-2021) also reviewed and approved the current study.

### Independent variables

#### Demographic factors

Age, sex, and years of education (for a further description, see Supplementary Information S2 and Fig. 1A).

#### Social determinants of health (SDH)

As defined by the World Health Organization, the SDH involve conditions in which people are born, work, live, and age, and the broader set of forces and systems shaping the needs of daily life [5, 53]. SDH can be characterized by different domains including socio-economic resources, social adversities, social participation and social context factors associated with pathological aging [53] (Supplementary Information S3 and Fig. 1A).

#### - Socio-economic resources

This domain included (a) salary in monthly income, (b) housing type, (c) housing’s floor, (d) housing services, (e) lifetime occupation, (f) pension plan, (g) institutional health support, and (h) medication access (Supplementary Information S3).

#### - Social adversities

This domain considered (a) forced displacement by conflict or violence, (b) age of first displacement, (c) perceived discrimination experiences, (d) physical assaults, (e) violence experience, and (f) social isolation (Supplementary Information S3).

#### - Social participation

Four measures were reported, including (a) social group membership, (b) participation in social groups, (c) social volunteering activities, and (d) religiosity (Supplementary Information S3).

#### - Complementary social-context factors (CSCF)

This domain is comprised by (a) civil status, (b) living conditions, (c) area of residence, (d) race group identity, and (e) ethnic group identity.

#### Cardiometabolic factors (CMF)

This domain encompassed a self-report assessment of diabetes and cardiovascular risk. In addition, body mass index (BMI) was obtained by dividing the weight by the square of height, expressed in units of kg/m^2^ (Supplementary Information S4).

#### Physical and mental health conditions (PMHC)

We included the following complementary variables associated with SDH and CMF that impact aging trajectories for the data-driven approach.

#### - Medical conditions

This domain includes different measures associated with dementia [54–56], including (a) auditory exam, (b) vision exam, (c) self-report of physical symptoms in the last 30 days, (d) self-report of medical conditions in the last 15 days, and (e) history of fall(s) (Supplementary Information S4).

#### - Lifestyle factors

This domain included four primary measures previously associated with dementia risk [12] and CMF: (a) smoking, (b) alcohol consumption, (c) nutritional state, and (d) nutritional support (Supplementary Information S4).

#### - Mental health factors

This domain included five different measures previously associated with pathological aging and SDH [57]: (a) presence or absence of depressive symptoms, (b) history of mental disorders, (c) self-perception of aging, (d) fear of falling, and (e) sexual activity valuation (Supplementary Information S4).

#### Grip strength

Participants’ grip strength was included as it is a critical variable associated with frailty, functional status, and adverse outcomes in older adults [20, 58]. Grip strength was assessed using the average of two Takey hydraulic dynamometers (the Smedley Hand Dynamometer III) attempts, and we considered the stronger hand category measure for analyses [58] (Supplementary Information S5).

### Outcome variables

#### Cognition

The general cognitive functions were assessed using the Folstein Mini-Mental State Examination (MMSE), a validated international scale translated to Spanish [59]. MMSE is a classical instrument for assessing global cognition and involves five domains: verbal memory, working memory, language, visuospatial, and orientation functions. Following previous procedures, we used a cutoff point of 23 points or less as indicating a low cognitive level [59] (Fig. 1A).

#### Functional capacity

Functional capacity was measured using the Barthel scale [60] and Lawton and Brody functional scale [61]. In addition, we included a measure of functional mobility in the functional capacity measure, which was determined by measuring gait speed [62] (Fig. 1A).

#### - The Barthel index

This index weighs the patients’ difficulties in activities of daily living, including basic activities of everyday life, for example, self-maintenance skills (such as bathing, grooming, dressing, toilet use), continency habits (bowels and bladder continency), and mobility (i.e., transfers, use of stairs) [60]. Individuals are scored on ten activities that are summed to give a score ranging from 0 (totally dependent) to 100 (entirely independent). A cutoff point of 80 in the Barthel scale was used to determine daily life activities’ normal functioning. This measure has been shown to have higher sensitivity in capturing daily life functions [63] (Fig. 1A).

#### - The Lawton and Brody functional scale

This instrument evaluates the participant’s functional capacity for performing a group of eight instrumental activities that are needed to live independently in the community (phone use, shopping, food preparation, housekeeping, laundry, transport use, handling medicines, and management of money). A summary score ranges from 0 (low function, dependent) to 8 (high function, independent) for women and 0 to 5 for men (Fig. 1A). We followed this procedure of scoring following previous procedures [61].

#### - Functional mobility

We measured the participants' gait speed—a measure of mobility [62]. Previous studies have shown that gait speed is related to instrumental functionality and frailty [62]. To this end, participants were asked to walk 3 m at their regular pace two times from a standing position. We used the best of both trials to determine gait speed which was used as a continuous variable and also categorized as slow gait speed (defined as a value of ≤ 0.8 m/s according to the Colombian validation cutoff [64]) accordingly.

### Statistical approaches

#### Theory-driven analyses: multigroup-structural equation models

We implemented a multigroup-structural equation models approach (SEM) to estimate the causal relationships between groups of latent theoretical categories (demographic factors, SDH, CMF) measured with different observed variables and to characterize the degree to which these variables predicted cognition and functional status (Fig. 1B). The SEM is a hybrid statistical technique that includes confirmatory factor analysis, path analysis, and regressions to test the predictive models of different outcomes and estimate the causal relationships among variables [65].

Measured (observed) variables are restrained directly and were used and tested (using significant regressor scores as predictors) to build latent (unobserved) variables. To assess the importance of observed variables to create latent variables (and considering measured variables were binary, ordinal, or continuous and not normally distributed), we used diagonal weighted least squares (DWLS) with a mean-corrected statistic known as weighted DWLS (WLSM) [66]. We evaluated the goodness-of-fit of each model to the data via global model fit indices that adjust for nonnormality: the robust comparative fit index (the robust CFI [67]) and the robust root mean square error approximation (the robust RMSEA [67], Supplementary Information S6).

##### Latent variables, factorial invariance, and covariables

We created latent variables for each one of the outcome measures (cognition and functional capacity). Moreover, a latent variable for each domain of SDH, including a socio-economic resources factor (SDH-SE), a social adversities factor (SDH-SA), and social participation (SDH-SP), was designed. Then, we built a latent global SDH factor including all domains. High scores were indicative of poor SDH in those domains. For details and criteria for building latent variables and testing measurement invariance, see Table S2 and Fig. 1B. Furthermore, we created the latent CMF by including diabetes, body mass index, and a cardiovascular index. A higher CMF score is indicative of exhibiting increased cardiometabolic risk.

In the SEM, age and years of education were included as covariables. Regarding sex, invariance tests supported a scalar invariance model in which the latent variances were equal across sex (see Supplementary Information Table S2). Complementary information of SEM is provided in Supplementary Information S6. The multigroup-structural equation models were run in a subsample of *N* = 15,577—the number of individuals who had completed the total number of variables assessed. All data analyses were performed in RStudio [68], using various packages (including semTools (0.5–3), SEM Lavaan R package (0.5–12 (BETA)), and Tidyverse [69]).

#### Data-driven analyses: machine-learning methods

We followed machine learning procedures to track the weight of each factor (including demographics, SDH, CMF, and a group of additional social and medical factors-physical and mental health conditions) in determining cognition and functional status. In our study, subjects who exhibited an MMSE score of fewer than 23 points were considered as having low cognitive functioning and above 24 points as having high cognitive function [59]. Moreover, individuals with scores below 80 points in Barthel were labeled as a having disability in daily life activities, and those who scored above 80 as having adequate daily life activities [70] (Fig. 1B).

Our machine learning approach included different steps. First, we ran feature elimination and stabilization using a k-fold scheme (*k* = 10). Second, we used the XGBoost [27] classifier for cognitive and functional levels. The XGBoost algorithm is a gradient boosting machine (GBM) implementation that provides parallel computation tree boosting, enabling fast and accurate predictions and advanced regularization techniques to avoid overfitting [71]. Third, the XGBoost was fitted by using several hyperparameters, such as the learning rate, the minimum loss reduction required to partition further a leaf node, the maximum depth of a tree, the maximum number of leaves, and the regularization weights. To choose the best parameters for the classification in this high dimensional hyperparameter space, we used Bayesian optimization [72]. Following best practices in machine learning [33], we employed a k-fold validation approach (k = 10) using 80% of the sample for training; and 20% for testing in an out-of- sample validation (Supplementary Information S7 and Fig. 1B). We tested the steps mentioned above using all possible predictors of cognition and functionality. Moreover, after selecting the best 25 predictors of cognition and functional capacity, we ran a second group of analyses to assess the predictive scores of those predictors. All models were run using machine XGBoost libraries in Python.

## Results

### Theory-driven analyses (SEM)

The implementation of SEM was aimed to assess associations between different variables and outcomes (latent variables) of different demographic, SDH, and medical factors. SEM models reached good statistical parameters supporting significant associations between different latent variables and outcomes with high goodness-of-fit indexes (Supplementary Information S8).

### Cognition

**Fig. 2:**
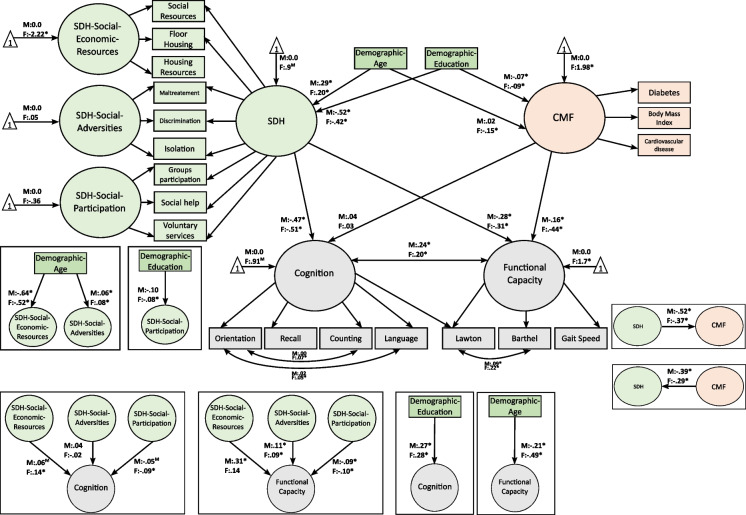
Structural equation modeling of the impact of inequities on cognitive and functional aging. **A** The SEM of cognition and functionality assessing different DG, SDH, and CMF predictors. **B** The two left images shows the specific predictive values of each of the SDH domains (SDH-socio-economic resources, SDH-social adversities, and SDH-social participation) on cognition and functionality. **B** The two right images revealed the prediction values of age and years of education on cognition and functionality. For identification of the model, factor means were fixed to zero in males (“M”) and freely estimated for females (“F”). These estimated factor means are expressed as standard deviation units (SD). For example, the factor mean for CMF indicates that the females scored 1.58 SD higher than males (*p* < 0.001); *significant effects or sex differences in factor means (*p* > 0.05). For simplicity, residual variances and observed intercepts are not shown in the figure. SDH, global social determinants of health factor; SDH-SE, social determinants of health-socio-economic resources; SDH-SA, social determinants of health-social adversities; SDH-SP, social determinants of health-social-participation; Education, years of education; CMF, cardiometabolic factors

SEM showed that individuals with high SDH (*M*: − 0.47, *P* < 0.001; *F*: − 0.51, *P* < 0.001) and poor socio-economic resources (*M*: − 0.06, *P* = 0.098; *F*: − 0.14, *P* < 0.01) exhibited low cognition. Moreover, high education (*M*: 0.27, *P* < 0.01; *F*: 0.28, *P* < 0.01) and high social participation (*M*: 0.09, *P* < 0.05; *F*: 0.05, *P* < 0.05) predicted elevated cognitive scores. The presence of social adversities and CMF factors did not show significant effects on cognition (Fig. 2).

### Functionality capacity

SEM revealed that individuals with global negative SDH (*M*: − 0.28, *P* = 0.001; *F*: − 0.31, *P* < 0.01), increased social adversities (*M*: 0.11, *P* < 0.01; *F*: 0.09, *P* < 0.01), low socio-economic resources (*M*: − 0.31, *P* = 0.001; *F*: − 0.14, *P* < 0.001), and reduced social participation (*M*: − 0.09, *P* < 0.01; *F*: − 0.11, *P* < 0.01) exhibited low functional capacity. Moreover, older individuals (*M*: − 0.21, *P* < 0.001; *F*: − 0.43, *P* < 0.001) with high-risk CMF (*M*: − 0.16, *P* < 0.001; *F*: − 0.44, *P* < 0.001) showed low functional capacity (Fig. 2).

### Complementary results

Results on cognition and functionality remained similar when the main predictors were analyzed independently. No mediation effects of high-risk CMF on the association between SDH and cognition and functionality were observed. Also, no mediation effects were detected when we analyzed the effects of global SDH factor on the association between high-risk and functionality (Supplementary Information S8, Supplementary Figs. 1–4).

### Data-driven results (machine learning)

The machine learning models assess the extent to which the combination of multiple factors relates to brain health outcomes. Appropriate classification for accuracy of cognition and functionality was obtained with the full set of predictors and a subset of predictors with higher scores. Moreover, the machine learning models helped to weigh the most relevant predictors of cognition and functionality (Supplementary section S7) (Table 1).

**Table 1:** SEM testing sequence for measurement invariance across sex

Model	*SB* χ^2^	*df*	*p*	Robust CFI	Robust ΔCFI	Robust RMSEA
Multidimensional model (invariance models testing sequence for sex)						
Configural	2294.944	312	.000	.916	-	.027 [.026–.028]
Metric	2382.970	334	.000	.909	.007	.027 [.026–.028]
Scalar	2447.567	340	.000	.908	.001	.027 [.026–.028]
Scalar-variance	2469.460	347	.000	.907	.001	.027 [.026–.028]

The 90% confidence intervals of the robust RMSEA are given in brackets. Scalar-variance: a scalar invariance model with latent variances constrained to be equal across sex

ΔCFI, CFI differences; *df*, degree of freedom; *p*, p value; Robust CFI, the robust comparative fit index; Robust RMSEA, the robust root means square error approximation; *SB* χ^2^, the Satorra-Bentler correction factor for the chi-square statistics

#### Cognition

An appropriate classification accuracy was obtained with the full set of predictors as well as with a subset of twenty-five predictors with the higher scores. Before optimization, with the total number of variables, the classification model presented an AUC = 0.76 and accuracy = 0.75 (sensitivity = 0.72, specificity = 0.79, the precision = 0.80, F1 = 0.79, and recall = 0.74). After feature optimization, stabilization was achieved for the XGBoost classification reaching an AUC = 0.87 and accuracy of 0.87 (sensitivity = 0.84, specificity = 0.85, precision = 0.83, F1 = 0.88, and recall = 0.79) with the twenty-five predictors included in the model (Table 2). The machine learning models showed that the top predictors of low cognitive level were older age, low years of education, antecedents of hypertension, smoking, poor institutional help, vulnerable urban background, self-perception of being older, and black and white race/ethnic groups (Table 2).

**Table 2:** Best predictors of low cognitive level as measured by machine learning methods listed in order (e.g., from the strongest to the weakest)

Predictors of low cognitive level	Type of subfactor and category of predictors of low cognitive level
Age	Demographic factors
Years of education	Demographic factors
Hypertension	CMF
Sex	Demographic factors
Smoking	Lifestyle subfactor-PMHC
Poor institutional help	Socio-economic resources-SDH
Type of housing	Socio-economic resources-SDH
High self-perception of aging	Mental health subfactors-PMHC
No self-defined ethnicity	Ethnicity subfactor-CSCF
White race self-perception	Race subfactor-CSCF
Black race self-perception	Race subfactor-CSCF
Poor economic resources	Socio-economic resources-SDH
Black ethnic identification	Ethnicity subfactor-CSCF
Witness’ violence	Social adversities-SDH
Poor floor conditions	Socio-economic resources-SDH
Social isolation	Social adversities-SDH
High maltreatment experiences	Social adversities-SDH
Fear of falling	Mental health subfactors-PMHC
Vision functioning	Lifestyle subfactor-PMHC
Moderated maltreatment experiences	Social adversities-SDH
Reduced institutional help	Socio-economic resources-SDH
Low self-perception of aging	Mental health subfactor-PMHC
Health problems (last 15 days)	Medical conditions subfactor-PMHC
Housing conditions	Socio-economic resources-SDH
Employment	Occupation subfactor-CSCF

CMF, cardiometabolic factors; SDH, social determinants of health; PMHC, physical and mental health conditions; CSCF, complementary social context factors

#### Functional capacity

Non-optimized, full predictors results yielded an AUC = 0.79 and an accuracy of 0.81 (sensitivity = 0.73, specificity = 0.81, precision = 0.83, F1 = 0.81, recall = 0.76). After optimization, the classification with the best twenty-five predictors yielded an AUC = 0.942 and accuracy of 0.93 (sensitivity = 0.92, specificity = 0.95, precision = 0.91, F1 = 0.90, and recall = 0.81) (Table 3). Machine learning models were found as top predictors of low functionality level, low gait speed, a high body mass index, older age, low employment, low grip force, and mental health antecedents (Table 3).

**Table 3:** Best predictors of low functionality level as measured by machine learning methods listed in order (e.g., from the strongest to the weakest)

Predictors of low functionality level	Type of subfactor and category of predictors of low functionality level
Speed of waking	Physical subfactors-PMHC
Body mass index	CMF
Age	Demographic factors
Employment	Occupation subfactor-CSCF
Grip force	Physical subfactor-PMHC
Poor institutional help	Socio-economic resources-SDH
Mental disease antecedents	Mental health subfactor-PMHC
Falls	Mental health subfactor-PMHC
Smoking antecedents	Lifestyle subfactors-PMHC
Moderate institutional help	Socio-economic resources-SDH
Widow	Marital status-CSCF
Hypertension	CMF
Housing in urban area	Socio-economic resources-SDH
Fear of falling	Mental health subfactor-PMHC
Diabetes	CMF
Housing	Socio-economic resources-SDH
Stroke	CMF
Health problems (las 30 days)	Medical conditions subfactor-PMHC
Mild fear of falling	Medical conditions subfactor-PMHC
Health problems (last 15 days)	Medical conditions subfactor-PMHC
Absence of institutional help	Socio-economic resources-SDH
Poor economic resources	Socio-economic resources-SDH
High medication’s consumption	Medical conditions-PMHC
Auditory functioning	Lifestyle subfactor-PMHC
Religious participation	Social participation-SDH

CMF, cardiometabolic factors; SDH, social determinants of health; PMHC, physical and mental health conditions; CSCF, complementary social context factors

### Complementary analyses

#### Linear regression model predicting cognition

We ran a linear regression using the MMSE as outcome, and demographic factors, SDH, medical factors, and CMF as independent variables. The model reached significant values (*F* (1, 23,693) = 0.30, *p* = 0.01, *η*^2^ = 0.06) with the following significant predictors: mental problems, living alone, education falls, house conditions, age, alcohol consumption, and smoking. No significant effects were observed for CMF, physical activity, or sex (see Table S3).

#### Linear regression model predicting daily life functionality

The Lawton and Brody scale was considered as outcome, and the demographic factors, SDH, medical factors, and CMF as independent variables. The model was significant (*F* (1, 23,693) = 0.51, *P* = 0.01, *η*^2^ = 0.06) and revealed all factors except education reached significance (see Table S4).

## Discussion

In this population-based study, we used theory- and data-driven methods to assess whether factors associated with inequity (SDH, CMF, and complementary social and medical factors) accurately predict cognition and functional capacity in aging in an underrepresented LMIC population (Colombia). Results showed that a combination of SDH, CMF, demographics, and additional social and medical factors contribute to reach high prediction values of cognitive and functional capacity. According to the theory-driven results, SDH and education were the best predictors of cognition. The data-driven results confirmed the importance of SDH (70% of top predictors) in predicting cognition, followed by age, education, gender, hypertension, smoking, and mental health factors. In contrast, a distributed combination of SDH, CMF, physical and demographic factors predicted functional capacity as revealed by the theory-driven results. The data-driven results (machine learning XGBoost classifier) confirmed the importance of SDH (50% of top predictors) in forecasting functional capacity followed by physical factors (speed gait, force of grip), medical factors (including CMF), and age. Thus, this population-based study combined theory-driven and machine-learning approaches to identify critical predictors of cognition and functional capacity in older adults in Colombia. Taken together, results revealed a more significant role for SDH than CMF and other medical factors in determining cognition and (at a lesser extent) functional capacity.

### The impact of inequity signatures on cognition

**Fig. 3:**
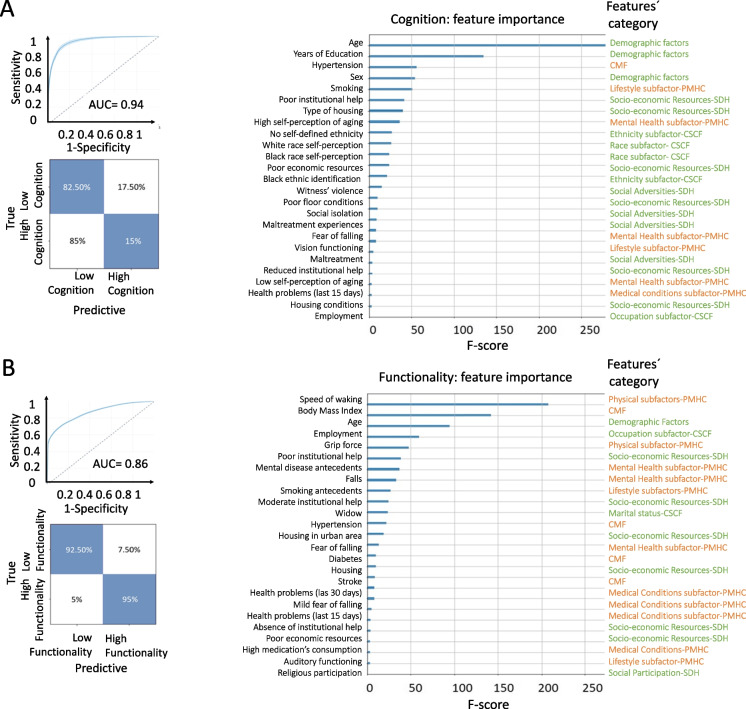
Data-driven prediction of cognitive and functional outcomes. **A** The machine learning results of cognitive prediction. In the left side, we showed the ROC curve of cognition prediction indicating specificity (true positive rate) and sensitivity (false positive rate) and the area under the curve (AUC = 0.94). On the right side is a feature importance plot of the most relevant *predictors* for the classification organized in the three big categories (SDG, SDH, and PMHC). **B** The machine learning results of functional prediction. In the left side, we presented ROC curve of functional capacity prediction indicating specificity (true positive rate) and sensitivity (false positive rate), while calculating the area under the curve (AUC = 0.86). SDG, socio-demographic conditions; SDH, social determinants of health; SDH-SE, social determinants of health-socio-economic resources; SDH-SA, social determinants of health-social adversities; SDH-SP, social determinants of health-social participation; Education, years of education; PMHC, physical and mental health conditions; PMHC-MSC, physical and mental health conditions-medical somatic conditions; PMHC-EF, physical and mental health conditions-lifestyle factors; PMHC-MH, physical and mental health conditions-mental health factors; PMHC-MB, physical and mental health conditions-motor and physical markers

The assessment of predictors of cognition provides important insights. Previous studies in HICs and LMICs have revealed a preeminence of medical factors over social ones in predicting cognition [12, 13]. The Lancet Commission on Dementia prevention, intervention, and care report in 2020 [12] has indicated a high relative risk of education, CMF, traumatic brain injury, lifestyle factors (including smoking and alcohol consumption), hearing problems. Social factors have been only indicated to a secondary extent (17). Our results add novel evidence from an underrepresented LMIC population, highlighting the role of SDH (mainly social resources and social participation) over medical factors as determinants of cognition. Moreover, additional data-driven analyses revealed that other social factors, including ethnic factors, experiences of forced displacement due to violence, and maltreatment were among the twenty-five best predictors. The increased presence of social stress due to different stressors, social adversities, and ethnic disparities have been associated with cognitive decline [73]. Our results revealed that the presence of pervasive social disadvantages and adversities represents a strong predictor of brain health and could increase the impact of SDH over CMF as direct predictors of brain health outcomes (Fig. 3).

Unlike studies in HICs [43]**,** our theory-driven analyses did not replicate the effects of CMF on cognition. Instead, our results confirmed a previously hypothesized differential pattern in LMICs [74, 75] characterized by a greater impact of SDH than CMF and other related physical/mental health factors on cognition. In particular, the direct impact of SDH on CMF could explain the absence of association between CMF and cognition (Fig. 2). Supplementary analyses revealed significant direct effects (CMF cognition; SDH cognition) when we analyzed those factors independently (Supplementary Figs. 3 and 4). Thus, results suggest that complex interactions between social and medical factors shape cognitive levels in our sample. In addition, hypertension, smoking, and mental health factors appeared to be significant predictors of cognition in the data-driven analyses. Hypertension [76], smoking [77], and mental health factors—particularly depression and anxiety symptoms [78, 79]—have been independently associated with cognitive maintenance and decline in previous studies.

Medical factors such as diabetes [80] or alcohol consumption [81] have been identified as risk factors of cognitive decline in HICs and LMICs. However, these factors did not reach typical significance thresholds in our study. Once again, their possible direct effects on cognition could be attenuated when considering the weight and interactions of other factors related to SDH and CMF, particularly in LMIC settings whose experience of transitioning to a higher burden of non-communicable diseases is more recent [10]. Further studies also demonstrated a lower relative risk of diabetes and alcohol compared to hypertension or obesity [12, 13]. Thus, in our research, these factors did not reach a significant impact when integrated with more robust predictors such as SDH.

Concerning demographics, age did not have a direct effect on cognition in the theory-driven analyses, although it was a critical predictor in data-driven analyses, with the most significant association observed in women. The multiple interactions between factors associated with cognition and the high impact of SDH on cognition could shadow the direct association of age on cognition. Regarding gender, both theory-driven and data-driven results revealed worse cognitive levels in women as previously reported [82]. In Colombia and other LMICs, high gender inequity levels are observed, with women facing increased rates of living alone, poor social participation, and reduced opportunities for healthcare access due to imposed social and cultural norms [83]. Another significant theory-driven and data-driven predictor of cognition was education, as has been frequently reported [15, 84]. Expectedly, education was directly associated with SDH. Individuals from populations with high social disadvantages have difficulties accessing education [15] and have more impaired memory, abstraction, reasoning, and attention skills [84]. Education has also been associated with decreased cognitive activity, more comorbidities or poor physical health, and reduction of social interactions [82]. Thus, results confirmed that gender and education are important predictors of cognition in LMICs when other multiple predictors (including SDH and CMF) are analyzed.

### The impact of inequity on functional capacity

Our results provide new evidence of the impact of SDH and CMF and other medical factors on functional capacity. In line with previous studies, the theory-driven approaches yielded associations between functional capacity and cognition [85]. Contrary to reports from HICs [86], the SDH factors (including reduced social resources, increased social adversities, and reduced social participation) reached predictive values equivalent to those of medical factors in predicting functionality as revealed by both theory- and data-driven approaches. Moreover, the social disadvantages in Colombia may reduce the quality of health which then impacts mobility and physical autonomy, as reported in HICs [43, 87]. Similarly, the impact of physical and medical factors on functional capacity observed in theory- and data-driven analyses could be explained by reductions in mobility, general activity, and physical activity associated with medical diseases, including CMF. This pattern of results aligns with past studies revealing that physical functioning (a crucial factor of frailty) determines autonomy in daily life activities [20, 58] (Figs. 2 and 3).

Regarding the role of demographic factors on functional capacity, results converge with previous reports from HICs and LMICs showing that older adults exhibited reduced instrumental functionality [20, 58]. Moreover, gender modulated the associations between SDH and functional capacity, with a significant impact of SDH in women as previously reported [87]. Furthermore, education did not reach a direct association with functional capacity. However, we observed an indirect effect of education on cognition, SDH, CMF in the theory-driven analyses. These indirect effects could attenuate a direct association between education and functional capacity. This interpretation is supported by past studies showing a more direct impact of education on health status but an indirect effect of education on daily life activities [85]. Moreover, education is an essential factor associated with SDH [88] and CMF [89]. Our results highlighted the role of gender and age in predicting functionality in LMICs when multiple sources of inequity are considered.

Our results were also confirmed by classical regression models. The linear regression model using cognition as a dependent variable was predicted by demographic factors, SDH, and lifestyle factors. Moreover, in line with SEM and machine learning results, no associations were found between cognition and CMF (Table S3). Similar confirmation was observed for functionality, where the regression model revealed the same predictors (demographics, SDH, CMF, lifestyle, complementary medical, and social factors (Table S4).

Although the pattern of results could be affected by correlations between factors, and the number of contrasts and variables could increase the chance of obtaining spurious correlations, our results do not support these potential effects. The theory-driven analyses (structural equation modeling) uses latent variables that weigh potential relationships between variables and penalize collinearity and spurious interactions between factors [90]. The machine learning approach also includes procedures (hyperparameter tuning) to control bias in classification due to non-relevant associations. This procedure assesses the predictive scores of each variable while controlling for the weight of the rest of the variables[28]. Thus, the influence of associations between variables was independently controlled in both approaches.

### Limitations and further assessments

Despite being one of a few nationally representative data from LMICs assessing these complex relationships between SDH, CMF, and cognition, our study has notable limitations. As is the case with most studies in the field [18, 33, 88], self-report measures employed in this study could underestimate or overestimate other predictors due to recall bias. However, self-reported measures are frequently used to track social and medical factors in aging [10, 11, 39]. Moreover, we combined self-reported measures with standardized objective measurements (i.e., physical functions and body mass index). Both variables reached similar scores in the SEM and data-driven approaches, suggesting that self-report and standardized/experimental effects are at least comparable. In any case, future assessment of SDH and CMF should employ objective measurements (blood biomarkers to track CMF and standardized scales to track SDH).

Among the group of predictors, some factors can be considered direct exposures (i.e., a direct measure of a factor considered as a risk factor associated with an outcome) and others proxies/markers of an exposure (an indirect measure of an exposure potentially associated with an outcome) [91]. Sociodemographic factors, including age and sex, can be considered direct exposure measures. Similarly, depression symptoms (i.e., direct measurement of the presence of depressive symptoms in the individuals) was also considered a direct exposure. By contrast, we considered proxies of a measure of those factors based on self-reports of an exposure. These included SES, social isolation, adverse social experiences, and CMF. A potential limitation of our study is the lack of direct measures for critical risk factors. However, both data-driven and theory-driven approaches discarded potential biases in the selection of factors and in the predictors’ ranking (as both exposures and proxies reached equivalent predictive scores). Similarly, previous studies have revealed high predictive scores using similar data (see [18, 39, 52] and Table S1). Future studies should combine immediate measures and proxies of measures or, in other words, objective and subjective measurements and test the prediction strength of each type of factor.

Our study design did not allow us to know the extent to which a specific factor predicts a clinical change in cognition and functionality. However, in this study, we referred to the prediction scores of variables regarding cognition and functionality outcomes. This use of “prediction” is based on statistical inference and refers to the power of a variable (predictor) to predict another variable (outcome) [92]. This meaning should not be confounded with a longitudinal prediction of a future outcome. Future longitudinal studies are required to predict brain health changes.

In this study, we implemented an SEM, which allows for the measurement of the mediating role of a variable between a factor and an outcomes [26] and has been used to track mediation effects in brain health studies [25, 93]. SEM is a hybrid technique that includes confirmatory factor analysis, path analysis, mediation analyses, and regression for building and testing the models of different outcomes and estimates potential causal and mediation relationships among groups of variables [26, 90]. However, our approach should not be confounded with mediation results of longitudinal data. Future longitudinal studies will help to formulate causal inferences and to clarify which variables mediate the interplay between risk and protective factors and brain health outcomes.

The study included more features of SDH than CMF. Although this disbalance may affect the predictor selection, several reasons suggest that is not the case. A different set of predictors were observed for cognition and functionality, with differential predictive scores for each outcome variable. Moreover, the best predictors of functionality also revealed a more significant weight of medical factors over social factors, discarding a generalized bias in the selection of predictors.

Large samples can impact statistical significance. However, in theory-driven analyses, we rely on measures of fit that are less biased (we excluded the χ^2^ as this index is biased by large sample sizes [94]). The CFI and RMSE indicators, which are less biased by sample size, provided reliable effects. In data-driven analyses, k-fold cross-validation hyperparameter tuning and Bayesian optimization reduced overfitting and prediction bias [95]. Also, prediction accuracy in machine data-driven learning tends to decrease with increased sample size [96], in opposition to parametric models. Our sample size is large enough and performed with the recommended combined metrics (recall, F1, AUC, sensitivity, and specificity), guaranteeing the reliability of the prediction values [96]. Further studies need to extend similar analyses of SDH and medical interactions in other LMICs and then compare those results with findings in UMICs and HICs. Moreover, future prospective studies should confirm the effects observed in the present study.

We assessed the individuals who completed the initial screening of MMSE to reduce the chance of including individuals having dementia. To avoid those potential biases, participant with MMSE scores below 13 were excluded, which reached around 17% of the sample. However, even those individuals with scores below 13 in the MMSE could have undiagnosed dementia or other related conditions. Future studies should include participants with lowest scores of MMSE and proper negative confirmation of dementia.

The sample of our both studies was different. In the theory-driven analyses, we only ran the SEM with a sample of 15,577 individuals with complete information on all variables assessed. As expected, the database included missing data, as often occurred with other population-based studies [97]. Although the missing values could impact the results obtained, these were broadly distributed and did not affect specifically any variable, as all models were run using individuals with more than 70% of all tested variables (Supplementary Information S6). However, future population-based studies should include additional strategies to assess and account for missing data in the results.

## Conclusions

The present results derived from a large population-based national survey in a LMIC revealed that different inequity signatures related to SDH and medical factors are crucial to predict brain health outcomes in aging. In contrast to findings from studies conducted in HICs, our results point towards different combinations of factors (SDH, CMF, and others) in predicting cognition and functional capacity. Convergent analysis suggests a hierarchy of predictors of healthy aging where the SDH is the most relevant predictor, followed by the CMF, demographics, and other medical factors. Cognition was primarily determined by SDH, whereas a more balanced contribution of SDH, medical, physical, and demographic factors predicted functionality. Results consistently showed multiple interactions between inequity signatures in predicting brain health outcomes at a population level in LMIC settings. The combination of robust methods with an integrated approach that included a broad spectrum of factors, from wider societal to individual-level factors, afforded the elicitation, in context, of multiple predictors of cognitive and functional deficits. Our results should inform policies and programmatic interventions to counter dementia globally and in low-resource areas in both HICs and LMICs. Finally, our results may be relevant for future consideration in personalized risk detection and to inform tailored prevention programs to enhance brain health in LMICs accounting for contemporary challenges including the rising rates of forced displacement and others.

### Supplementary Information

Below is the link to the electronic supplementary material.

Supplementary file1 (DOCX 683 KB)

## Data Availability

Requests to access the data should be directed to the Colombian Ministry of Health (repositorio@minsalud.gov.co). The original Spanish study is *Salud**, **Bienestar & Envejecimiento* (SABE Colombia). The analysis plan is available from request to the corresponding authors.

## References

[1] Wang Y, Pan Y, Li H (2020). What is brain health and why is it important?. BMJ.

[2] Nichols E (2022). Estimation of the global prevalence of dementia in 2019 and forecasted prevalence in 2050: an analysis for the Global Burden of Disease Study 2019. Lancet Public Health.

[3] Manes F (2016). The huge burden of dementia in Latin America. Lancet Neurol.

[4] The Lancet N (2020). Disparities in neurological care: time to act on inequalities. Lancet Neurol.

[5] Marmot M, Allen JJ (2014). Social determinants of health equity. Am J Public Health.

[6] Kuiper JS (2015). Social relationships and risk of dementia: a systematic review and meta-analysis of longitudinal cohort studies. Ageing Res Rev.

[7] Sharp ES, Gatz M (2011). The relationship between education and dementia an updated systematic review. Alzheimer Dis Assoc Disord.

[8] Lund C (2018). Social determinants of mental disorders and the Sustainable Development Goals: a systematic review of reviews. Lancet Psychiatry.

[9] Steptoe A and Zaninotto P. Lower socioeconomic status and the acceleration of aging: An outcome-wide analysis. Proc Natl Acad Sci. 2020;117(26):14911-14917. 10.1073/pnas.1915741117 PMC7334539 32541023

[10] Miranda JJ (2019). Understanding the rise of cardiometabolic diseases in low-and middle-income countries. Nat Med.

[11] Kivimäki M (2019). Physical inactivity, cardiometabolic disease, and risk of dementia: an individual-participant meta-analysis. BMJ.

[12] Livingston G (2020). Dementia prevention, intervention, and care: 2020 report of the Lancet Commission. Lancet.

[13] Livingston G (2017). Dementia prevention, intervention, and care. Lancet.

[14] Parra MA (2021). Dementia in Latin America: paving the way toward a regional action plan. Alzheimers Dement.

[15] Gross AL (2015). Effects of education and race on cognitive decline: An integrative study of generalizability versus study-specific results. Psychol Aging.

[16] Prynn JE, Kuper H (2019). Perspectives on disability and non-communicable diseases in low- and middle-income countries, with a focus on stroke and dementia. Int J Environ Res Public Health.

[17] Castellanos-Perilla N (2020). Factors associated with functional loss among community-dwelling Mexican older adults. Biomedica.

[18] Morros-González E (2017). The elderly with diabetes and associated factors. SABE study, Bogotá, Colombia. Acta Med Col.

[19] Zijlmans JL (2021). The interaction of cognitive and brain reserve with frailty in the association with mortality: an observational cohort study. Lancet Healthy Longevity.

[20] Finkel D (2019). Functional aging index complements frailty in prediction of entry into care and mortality. J Gerontol: Series A.

[21] Salazar A (2021). Undermining Colombia's peace and environment. Science.

[22] Curcio CL (2019). Elderly and forced displacement in Colombia. Colomb Med (Cali).

[23] Camacho PA (2020). Self-reported prevalence of chronic non-communicable diseases in relation to socioeconomic and educational factors in Colombia: a community-based study in 11 departments. Glob Heart.

[24] Gomez F (2016). SABE Colombia: survey on health, well-being, and aging in colombia—study design and protocol. Curr Gerontol Geriatr Res.

[25] Santamaría-García H (2020). The role of social cognition skills and social determinants of health in predicting symptoms of mental illness. Transl Psychiatry.

[26] West SG, Taylor AB, Wu W (2012). Model fit and model selection in structural equation modeling. Handbook Struct Equation Model.

[27] Kaufmann T, van der Meer D, Doan NT, Schwarz E, Lund MJ, Agartz I, Alnæs D, Barch DM, Baur-Streubel R, Bertolino A, Bettella F, Beyer MK, Bøen E, Borgwardt S, Brandt CL, Buitelaar J, Celius EG, Cervenka S, Conzelmann A, Córdova-Palomera A, … Westlye LT. Common brain disorders are associated with heritable patterns of apparent aging of the brain. Nat Neurosci 2019;22(10):1617–1623. 10.1038/s41593-019-0471-7 PMC6823048 31551603

[28] Bzdok D, Altman N, Krzywinski M (2018). Points of significance: statistics versus machine learning. Nat Methods.

[29] Donnelly-Kehoe PA (2019). Robust automated computational approach for classifying frontotemporal neurodegeneration: Multimodal/multicenter neuroimaging. Alzheimer's Dement: Diagn, Assess Dis Monit.

[30] Santamaría-García H (2021). Uncovering social-contextual and individual mental health factors associated with violence via computational inference. Patterns (N Y).

[31] Braveman P, Gottlieb L (2014). The social determinants of health: it's time to consider the causes of the causes. Public Health Rep.

[32] Garcia-Cifuentes E (2022). Muscular function as an alternative to identify cognitive impairment: a secondary analysis from SABE Colombia. Front Neurol.

[33] Garcia-Cifuentes E (2020). The role of gait speed in dementia: a secondary analysis from the SABE Colombia study. Dement Geriatr Cogn Disord.

[34] Miu J (2016). Factors associated with cognitive function in older adults in Mexico. Glob Health Action.

[35] Fernández-Niño JA (2018). Work status, retirement, and depression in older adults: An analysis of six countries based on the Study on Global Ageing and Adult Health (SAGE). SSM Popul Health.

[36] Pérez-Sousa MÁ (2021). Role for physical fitness in the association between age and cognitive function in older adults: a mediation analysis of the SABE Colombia study. Int J Environ Res Public Health.

[37] O'Donovan G (2020). Education in early life markedly reduces the probability of cognitive impairment in later life in Colombia. Sci Rep.

[38] Marquez I (2022). Motoric Cognitive Risk Syndrome: Prevalence and Cognitive Performance. A cross-sectional study. Lancet Reg Health - Americas.

[39] Mejia-Arango S (2021). Socioeconomic disparities and gender inequalities in dementia: a community-dwelling population study from a middle-income country. J Cross Cult Gerontol.

[40] Larnyo E (2022). Examining the impact of socioeconomic status, demographic characteristics, lifestyle and other risk factors on adults' cognitive functioning in developing countries: an analysis of five selected WHO SAGE Wave 1 Countries. Int J Equity Health.

[41] Guerrero Barragán A, Lucumí D, Lawlor B. Association of Leisure Activities With Cognitive Impairment and Dementia in Older Adults in Colombia: A SABE-Based Study. Front Neurol. 2021;12:629251. 10.3389/fneur.2021.629251. 10.3389/fneur.2021.629251 PMC7956952 33732207

[42] Somrongthong R (2017). Influence of socioeconomic factors on daily life activities and quality of life of Thai elderly. J Public Health Res.

[43] Storeng SH, Sund ER, Krokstad S (2018). Factors associated with basic and instrumental activities of daily living in elderly participants of a population-based survey: the Nord-Trøndelag Health Study, Norway. BMJ Open.

[44] Mukadam N (2019). Population attributable fractions for risk factors for dementia in low-income and middle-income countries: an analysis using cross-sectional survey data. Lancet Glob Health.

[45] Stephan BCM (2020). Prediction of dementia risk in low-income and middle-income countries (the 10/66 Study): an independent external validation of existing models. Lancet Glob Health.

[46] Sosa AL (2012). Prevalence, distribution, and impact of mild cognitive impairment in Latin America, China, and India: a 10/66 population-based study. PLoS Med.

[47] Prina AM (2019). A review of the 10/66 dementia research group. Soc Psychiatry Psychiatr Epidemiol.

[48] Gomez F (2016). SABE Colombia: survey on health, well-being, and aging in colombia—study design and protocol. Curr Gerontol Geriatr Res.

[49] Kobayashi LC (2019). Cognitive function and impairment in older, rural south african adults: evidence from "Health and Aging in Africa: A Longitudinal Study of an INDEPTH Community in Rural South Africa". Neuroepidemiol.

[50] Ocampo-Chaparro JM (2019). Frailty in older adults and their association with social determinants of Health. SABE Colomb Study Colomb Méd.

[51] Borda MB (2015). Relationship between cognitive impairment and instrumental activities of daily living (IADL): sabe bogotá, colombia study. J Neurol Sci.

[52] Borda MG (2021). Body mass index, performance on activities of daily living and cognition: analysis in two different populations. BMC Geriatr.

[53] Aranda MP (2021). Impact of dementia: Health disparities, population trends, care interventions, and economic costs. J Am Geriatr Soc.

[54] Novella JL (2001). Measuring general health status in dementia: practical and methodological issues in using the SF-36. Aging (Milano).

[55] Davies-Kershaw HR (2018). Vision Impairment and Risk of Dementia: Findings from the English Longitudinal Study of Ageing. J Am Geriatr Soc.

[56] Loughrey DG (2018). Association of age-related hearing loss with cognitive function, cognitive impairment, and dementia: a systematic review and meta-analysis. JAMA Otolaryngol–Head Neck Surg.

[57] Byers AL, Yaffe K (2011). Depression and risk of developing dementia. Nat Rev Neurol.

[58] Massy-Westropp NM (2011). Hand Grip Strength: age and gender stratified normative data in a population-based study. BMC Res Notes.

[59] Folstein MF, Folstein SE, McHugh PR (1975). "Mini-mental state". A practical method for grading the cognitive state of patients for the clinician. J Psychiatr Res.

[60] Mahoney FI, Barthel DW (1965). Functional evaluation: the Barthel index. Md State Med J.

[61] Lawton MP, Brody EM (1969). Assessment of older people: self-maintaining and instrumental activities of daily living. Gerontologist.

[62] Mehmet H, Robinson SR, Yang AWH (2020). Assessment of Gait Speed in Older Adults. J Geriatr Phys Ther.

[63] Törnquist K, Lövgren M, Söderfeldt B (1990). Sensitivity, specificity, and predictive value in Katz's and Barthel's ADL indices applied on patients in long term nursing care. Scand J Caring Sci.

[64] Gómez JF (2013). Validity and reliability of the Short Physical Performance Battery (SPPB): a pilot study on mobility in the Colombian Andes. Colomb Med (Cali).

[65] Hoe SL (2008). Issues and procedures in adopting structural equation modelling technique. J Quant Methods.

[66] Cheung GW, Rensvold RB (2002). Evaluating goodness-of-fit indexes for testing measurement invariance. Struct Equ Model.

[67] Savalei V (2018). On the computation of the RMSEA and CFI from the mean-and-variance corrected test statistic with nonnormal data in SEM. Multivar Behav Res.

[68] R Core Team R, Team RC. R: A language and environment for statistical computing. Vienna: R Foundation for Statistical Computing; 2018.

[69] Jorgensen TD, Pornprasertmanit S, Schoemann AM, Rosseel Y, Miller P, Quick C, Garnier-Villarreal M. semTools: Useful tools for structural equation modeling. R package version 0.5, 1; 2018.

[70] Yi Y, Ding L, Wen H, Wu J, Makimoto K, Liao X (2020). Is Barthel index suitable for assessing activities of daily living in patients with dementia?. Frontiers in Psychiatry.

[71] Torlay L, Perrone-Bertolotti M, Thomas E, Baciu M (2017). Machine learning-XGBoost analysis of language networks to classify patients with epilepsy. Brain Inf.

[72] Feurer M, Hutter F. Hyperparameter Optimization. In: Hutter F, Kotthoff L, Vanschoren J, editors. Automated machine learning. The springer series on challenges in machine learning. Cham: Springer; 2019. p. 3–33.

[73] Majoka MA, Schimming C (2021). Effect of social determinants of health on cognition and risk of Alzheimer disease and related dementias. Clin Ther.

[74] Houle B (2019). Cognitive function and cardiometabolic disease risk factors in rural South Africa: baseline evidence from the HAALSI study. BMC Public Health.

[75] Oi K, Haas S (2019). Cardiometabolic risk and cognitive decline: The role of socioeconomic status in childhood and adulthood. J Health Soc Behav.

[76] Liu L (2022). Association between blood pressure levels and cognitive impairment in older women: a prospective analysis of the Women's Health Initiative Memory Study. Lancet Healthy Longevity.

[77] Nyberg ST (2020). Association of Healthy Lifestyle With Years Lived Without Major Chronic Diseases. JAMA Intern Med.

[78] Semkovska M (2019). Cognitive function following a major depressive episode: a systematic review and meta-analysis. Lancet Psychiatry.

[79] Ismail Z (2017). Prevalence of depression in patients with mild cognitive impairment: a systematic review and meta-analysis. JAMA Psychiat.

[80] Hassing LB (2004). Type 2 diabetes mellitus contributes to cognitive decline in old age: A longitudinal population-based study. J Int Neuropsychol Soc.

[81] Sabia S (2014). Alcohol consumption and cognitive decline in early old age. Neurology.

[82] Salthouse TA (2009). When does age-related cognitive decline begin?. Neurobiol Aging.

[83] Angrisani M, Lee J, Meijer E (2020). The gender gap in education and late-life cognition: Evidence from multiple countries and birth cohorts. J Econ Ageing.

[84] Kremen WS, et al. Influence of young adult cognitive ability and additional education on later-life cognition. Proc Natl Acad Sci. 2019;116(6):2021-2026. 10.1073/pnas.1811537116 PMC6369818 30670647

[85] Lövdén M (2020). Education and cognitive functioning across the life span. Psychol Sci Public Interest.

[86] Ghaffari A, Rostami HR, Akbarfahimi M (2021). Predictors of instrumental activities of daily living performance in patients with stroke. Occup Ther Int.

[87] Tomioka K, Kurumatani N, Hosoi H (2017). Age and gender differences in the association between social participation and instrumental activities of daily living among community-dwelling elderly. BMC Geriatr.

[88] Kollia N (2018). Social determinants, health status and 10-year mortality among 10,906 older adults from the English longitudinal study of aging: the ATHLOS project. BMC Public Health.

[89] Rodrigues JAL (2020). Cardiometabolic risk factors associated with educational level in older people: comparison between Norway and Brazil. J Public Health.

[90] Harring JR, Weiss BA, Li M (2015). Assessing spurious interaction effects in structural equation modeling: a cautionary note. Educ Psychol Meas.

[91] Lee TA, Pickard AS. Exposure definition and measurement. Developing a protocol for observational comparative effectiveness research: a user's guide, Agency for Healthcare Research and Quality (US). 2013. 23469377

[92] Clauset A, Larremore DB, Sinatra R (2017). Data-driven predictions in the science of science. Science.

[93] MacKinnon DP, Fairchild AJ, Fritz MS (2007). Mediation analysis. Annu Rev Psychol.

[94] Vandenberg RJ (2006). Introduction: statistical and methodological myths and urban legends:where, pray tell, did they get this idea?. Organ Res Methods.

[95] Poldrack RA (2017). Scanning the horizon: towards transparent and reproducible neuroimaging research. Nat Rev Neurosci.

[96] MacDonald D (2000). Automated 3-D extraction of inner and outer surfaces of cerebral cortex from MRI. Neuroimage.

[97] Bolland AC, Tomek S, Bolland JM (2017). Does missing data in studies of hard-to-reach populations bias results? Not necessarily. Open J Stat.

