# Supplementary material for: The impacts of social determinants of health and cardiometabolic factors on cognitive and functional aging in Colombian underserved populations
Source: GeroScience. 2023 Feb 28;45(4):2405–23. doi: 10.1007/s11357-023-00755-z (PMC10651610; doi:10.1007/s11357-023-00755-z)
Supplement: Supplementary file 1 — Supplementary file1 (DOCX 683 KB) [file 11357_2023_755_MOESM1_ESM.docx]

**Supplementary Information**

**S1. Literature review of predictors of brain health**

**Searching criteria for cognition prediction in LMICs.**

*Cognition OR cognitive functioning OR cognitive decline OR cognitive screening OR dementia OR neurocognitive disorders OR mild cognitive impairment AND prediction OR prediction OR predictive factor OR risk factor OR protective factor OR sex OR age OR education OR education level OR social determinants of health OR isolation OR social isolation OR socioeconomic status OR income OR poverty OR health access OR neighborhood conditions OR hypoacusia OR cardiometabolic risk OR cardiometabolic factors OR hypertension OR diabetes OR obesity OR sedentarism OR gait speed OR handgrip strength OR lifestyle OR physical activity OR nutrition OR diet OR smoking OR alcohol AND Low-and-middle-income countries OR Latin America OR Latin American OR underrepresented populations AND non-stereotypical sample (see Table S1).*

**Searching criteria for functionality prediction in LMICs.**

*Daily life activities OR basic activities OR functionality OR functional autonomy OR dementia OR neurocognitive disorders OR mild cognitive impairment AND prediction OR predictive factor OR risk factor OR protective factor OR sex OR age OR education OR education level OR social determinants of health OR isolation OR social isolation OR socioeconomic status OR income OR poverty OR health access OR neighborhood conditions OR hypoacusia OR cardiometabolic risk OR cardiometabolic factors OR hypertension OR diabetes OR obesity OR sedentarism OR gait speed OR handgrip strength OR lifestyle OR physical activity OR nutrition OR diet OR smoking OR alcohol AND Low-and-middle-income countries OR Latin America OR Latin American OR underrepresented populations AND non-stereotypical sample (see Table S1).*

**S2. Supplementary Methods: Independent variables. Socio-demographic factors (DG):** SDG included three different variables, including 1) sex (what is your sex?; Response options: female, male, other); 2) age (what is your age in completed years?); 3) years of study (how many years of education have you completed?)

**S3. Supplementary Methods. Social determinants of health (SDH).**

**Socio-economic resources- SDH:** This domain included five types of variables describing information on different social and economic resources such as a) salary in monthly income (what is your monthly salary?; response options: up to an amount near $250 dollars, up to 500$, up to 750$, up to 1250$, up to 1500$, more than 1500$); b) housing type (what type of housing do you live in?; response options: chalet, apartment, room in tenancy, other type of room, indigenous housing, other type of housing); c) the conditions of the housing´s floor (what is the predominant material of your housing´s floor?; response options: marble, wall-to-wall, carpet or rug tile, vinyl, tablet, brick, polished and lacquered wood, parquet coarse wood, board, plank, other vegetable, cement, gravel, sand or soil); d) housing resources (does your house have the following services?; response options: using yes/no; questions: electricity, aqueduct, potable water, and sewerage); e) lifetime occupation (what type of employee were you most of the time?; response options: worker or employee of a private company; government worker or employee; rural laborer or field laborer; employer, business owner; self-employed; unpaid worker; independent professional; pieceworker; domestic employee; other; f) pension (do you receive assistance?; response options: yes/no); g) institutional help (during the last year, did you receive support from any institution or organization?; response options: using yes/no; questions: social welfare service, aging center, house care, religious temple, rehabilitation center, palliative care, other types of organization, no received help from any institution; h) medication access (are you able to obtain the prescribed medications or remedies?; response options: always, sometimes, rarely, never, other).

**Social adversities- SDH:** This domain included five types of variables describing experiences of social adversities, including a) forced displacement (have you ever been displaced by armed conflict or violence in your life?; response options: yes, no, other); b) age of the first displacement (how old were you when you were first displaced?; response options: age in completed years of the first displacement); c) discrimination (did you ever feel rejected, discriminated against, mistreated?; response options: often, sometimes, rarely, never, no answer, other); d) physical assaults (have you ever suffered physical assaults?; response options: often, sometimes, rarely, never, no answer, other); e) violence experience (have you ever experienced or were witness of violence?; response options: often, sometimes, rarely, never, no answer, other); f) social isolation (have you ever felt isolated?; response options: often, sometimes, rarely, never, no answer, other).

**Social Participation-SDH:** Participants answered on three different social participation activities, including a) social partnership (is there a person who accompanies you to most of the social and/or recreational activities that you do?; response options: yes, no; other); b) participation in groups (in which of the following groups do you participate?; response options: using yes/no; questions: religious, sports, politic, cultural, community, ethnic, ecologic, aging, health, exercise, other groups); c) volunteering (during the last year, have you ever provided or participated in any service voluntarily to an organization?; response options: using yes/no questions: social well-being, aging centers, children centers, academic institutions, health centers, religious temple, no participated voluntarily in any organization, no response); d) religiosity (in terms of religion, do you consider yourself as?; response options: not religious, a little religious, very religious, does not respond).

**Complementary social-context factors (CSCF):** This group of factors encompasses five subfactors including 1) marital status (what is your marital status?; response options: married/with a partner, separated/widower, single); 2) living conditions (are you living alone?; response options: yes/no); 3) area of residence (in which area is your home?; response options: urban, rural, other); 4) race group identity (according to your racial identity, do you recognize yourself as?; response options: indigenous, black, mulatto, white, mixed-race, other option?); 5) ethnic group identity (according to your culture or community of origin, do you recognize yourself as?; response options: indigenous, gypsy, raizal of the San Andrés y Providencia archipelago, Palenquero of San Basilio, Black, Afro-descendant, or Afro-Colombian, other option).

**S4. Supplementary Methods: Cardiometabolic Factors (CMF):** This domain encompassed ten variables, including a) diabetes (has a doctor or nurse ever told you that you have diabetes, that is, high blood sugar?; response options: yes, no); b) cardiovascular risk (has a doctor or nurse ever told you that you have cardiovascular problems; response options: yes, no); and c) body mass index (body mass index was derived by dividing the weight by the square of height (expressed in units of kg/m²).

**S5. Supplementary Methods: Complementary measures of physical and mental health conditions (PMHC)**

**Medical conditions:** included different measures such as a) hypertension (has a doctor or nurse ever told you that you have hypertension?; response options: yes, no); b) stroke (has a doctor or nurse ever told you that you have had a stroke?; response options: yes, no); c) auditory problems (have you ever had hearing problems?; response options: yes, no, other); d) vision problems (have you ever had vision problems?; response options: yes, no, other); e) self-report of health state in the last 30 days (in general, how has your health been in the last 30 days?; response options: very good, good, regular, bad, very bad, does not respond, does not know); f) self-report of health state in the last 15 days (in general, how has your health been in the last 15 days?; response options: very good, good, regular, bad, very bad, does not respond, does not know); g) falls (in the last year have you fallen to the floor?; response options: yes, no, other).

**Lifestyle factors:** This domain included different significant measures, including a) smoking (do you smoke?; response options: yes, no; how old were you when you started smoking?; response options: completed years when started smoking); b) alcohol consumption (in the last month, on average, have you had alcoholic beverages, including beer, wine, brandy?; response options: yes, no; if positive response, they were also asked: how many days per week, on average, did you consume alcohol?; response options: less than one day per week, 2 to 3 days a week, 4 to 6 days a week, every day, did not consume, does not respond, does not know); c) self-perception of nutritional state (do you have an appropriated nutritional plan?; response options: yes, no); and d) nutritional support (do you follow a nutritional plan?; response options: yes, no).

**Mental health factors:** Four different measures were included to assess mental health, including a) presence or absence of depressive symptoms (have you felt depressed in the last year; response options: yes/no, other); b) history of mental disorders (have you ever been diagnosed with a mental disorder? response options: yes, no, other); c) self-aging perception (do you consider yourself as older?; response options: yes, no, other); and d) importance of sexual activity (to what extent do you consider your sexuality as necessary in your life?; response options: critical, necessary, neither very important nor unimportant, less critical, nothing important, does not respond, other); e) fear of falling (do you have a fear of falling?; response options: very much, sometimes, slight, none, other).

**Physical functioning:** Participants’ grip strength was measured. Grip strength was assessed using the average of two Takey hydraulic dynamometers (the Smedley Hand Dynamometer III) attempts, and the more robust hand category measure was included for analyses[1].

**S6. Supplementary Methods: Structural equation model.**

The SB χ2 test examines the exact-fit hypothesis that there is no difference between the model-implied covariance matrix and the population covariance matrix. A non-significant p-value (p ≥ 0.05) supports the exact-fit hypothesis. A problem with the χ2 test is that as the sample increases, its power to detect differences between the model-implied covariance matrix and the population covariance matrix approaches one [2]. Previous studies have revealed that the χ2 test is highly determined by sample size [3, 4]. Therefore, given the sample size of the present study, we did not expect a non-significant p-value. For this reason, we have used other indexes to test the modeling fit, including the robust CFI and the robust RMSEA. The former assesses how the specified model improves fit over the null model (values > 0.90 considered as an acceptable fit, and values > 0.95 considered as good fit[5]). The robust RMSEA is an absolute fit index where a value of zero supports the exact-fit hypothesis (values > 0.08 are considered as poor fit, values in the range of 0.05-0.08 are considered as a good fit, and values ≤ 0.05 support the close-fit hypothesis [6]). Additionally, the strength of regression paths (i.e., effect sizes) among manifest and latent variables around -±.10, ±.30, and ±.50 was considered small/low, medium/moderate, and large/strong, respectively[7].

We tested three increasingly restrictive hypotheses about measurement invariance across sex: configural invariance, metric, and scalar invariance. The configural invariance hypothesis assumes that the number of factors and the correspondence between factors and the measured variables is the same across sex. In this model, all parameters are freely estimated in each group (except those used to identify the factor structure). If the configural invariance model fits the data well, the next step involves examining a model with unstandardized regression weights (aka, factor loadings) being invariant across groups (i.e., metric invariance). The scalar invariance model assumes metric invariance and requires equal unstandardized intercepts (i.e., the unstandardized mean of measured variables) over the groups. We used a value of the robust ΔCFI (i.e., differences) smaller than 0.01 in a successive comparison of models to confirm that the null hypothesis of invariance should not be rejected (i.e., configural invariance vs. metric invariance, and metric invariance vs. scalar invariance [8]). Furthermore, to support measurement invariance, the robust RMSEA value of the model with increasing constraints (e.g., metric invariance) should fall within the 90% CI of the less restrictive model (e.g., configural invariance [5]). Retaining the metric invariance hypothesis enables formal comparisons of the variance-covariance of factors across groups. A scalar factorial invariance is a precondition for comparing latent factor means across groups and indicates that the latent variables have a common factorial structure across sex [9]. All data analyses were performed in RStudio [10], using various packages (including semTools (0.5-3), SEM Lavaan R package (0.5–12 (BETA)), and Tidyverse[11-14]). Although we assessed the Satorra-Bentler correction factor for the chi-square statistics (SB χ2), we expect significant χ2 values due to the larger sample size. Previous studies have revealed high chances of obtaining a statistically significant chi-square in large sample sizes where there may only be a trivial misfit [2, 9, 15, 16]. Considering these pieces of evidence, goodness-of-fit of each model to the data was evaluated via global model fit indices that adjust for nonnormality: the robust comparative fit index (the robust CFI [17]) and the robust root mean square error approximation (the robust RMSEA [17]).

All models were run using individuals with more than 70% of complete values in all tested variables. Table 1 reports absolute and relative fit indices for testing measurement invariance across sex. As expected, the SB χ2 for all measurement invariance models showed a significant p-value (see Table 1). However, the other fit indices supported the fit of each model to the data (see robust CFI and RMSEA in Table 1).

Then we tested whether latent variances were equal across sex (i.e., scalar-variance model). As Table 1 displays, the robust CFI showed an acceptable fit of the configural invariance model to the data suggesting that, across sex, the same factors were manifested by the same measured variables. Also, the metric invariance model revealed an acceptable fit to the data (see Table S2). The robust ΔCFI value was not greater than 0.01, and the robust RMSEA value fell within the 90% CI of the configural invariance model. Thus, the metric invariance model supports the hypothesis that the latent variables were manifested in the same manner in each group; specifically, the unstandardized estimates of the factor loadings were equal across sex. The scalar invariance model fits the data well (see robust CFI and robust RMSEA in Table 1), the robust ΔCFI between the metric invariance model and the scalar model was not larger than 0.01, and the robust RMSEA of the latter model fell within the 90% CI of the former. The goodness-of-fit indices of the scalar-variance model were adequate, the robust CFI was acceptable, and the robust RMSEA retained the exact fit hypothesis.

Furthermore, the robust ΔCFI between the scalar invariance and the scalar-variance models was not larger than 0.01. In contrast, the robust RMSEA of the latter model fell within the 90% CI of the former model. Taken together, these findings provide strong support for assuming a scalar invariance in which the latent variances were equal across sex.

All factor means were fixed to zero in males (“M”) and freely estimated in females (“F”; in Figure 2, see the parameters above the triangles pointing to each factor) in the model. Thus, factor means for SDH (values provided above the triangles pointing to the SDH factor) indicate that females marginally scored 0.89 standard deviations higher than males (p = 0.095). Relative to males, females performed higher in cognition (F = 0.90, p = 0.064) and significatively better in functionality (F = 1.7, p > 0.05). In the domain of the SDH-Socio-economic resources, females scored -2.19 standard deviations below males, while in the CMF factor, females scored 1.58 standard deviations above males (see Figure 2).

**S7. Supplementary Methods: Data-Driven Methods. Machine-learning procedures.** We followed different machine learning procedures to track the best predictors of two outcomes (cognition and functional capacity). In our study, an individual who exhibited an MMSE score of fewer than 23 points was considered as having low cognitive functioning following previous studies [18-20]. Additionally, an individual with scores below 80 points in Barthel was labeled as having a low functional level as previously reported [21].

**Feature elimination and stabilization:** We performed a progressive feature elimination to select the optimum predictors after stabilization [22] using a k-fold scheme (k=10). We started the feature optimization process with 63 predictors. We used the Gini scores to eliminate elements by removing predictors with the lowest importance at each iteration and checked for the robustness of our results based on the final number of predictors after stabilization for both samples [23]. Finally, we kept the N as first predictors in the ranking, where N is the optimal number of predictors such that using more than N predictors fails to improve the classifier’s performance [23].

**Classification:** We used the XGBoost [24] classifier for cognitive and functional levels. The XGBoost algorithm is a Gradient Boosting Machines (GBM) implementation that provides parallel computation tree boosting, enabling fast and accurate predictions and advanced regularization techniques to avoid overfitting [25]. This algorithm has been proven successful in several diagnostic applications [26-28]. GBMs are based on the gradient boosting technique, in which ensembles of decision trees iteratively attempt to correct the classification errors of their predecessors by minimizing a loss function (i.e., a function representing the difference between the estimated and true values) pointing in the negative gradient direction [29]. Compared to other GBM algorithms, XGBoost provides regularized boosting, helping to reduce overfitting and thus providing more generalizable results [28, 30].

**Hyperparameter tuning and data partition:** The XGBoost has several hyperparameters, such as the learning rate, the minimum loss reduction required to make a further partition of a leaf node, the maximum depth of a tree, the maximum number of leaves, and the regularization weights. To choose the best parameters for the classification in this high dimensional hyperparameter space, we used Bayesian Optimization [31, 32]. This state-of-the-art optimization framework demonstrated broad applicability to different problem settings. This iterative algorithm has two key ingredients: a probabilistic surrogate model and an acquisition function to decide which point to evaluate next. At each step, a new issue of the hyper-parameter space to explore is selected to be the maximum activation function of the prior knowledge and the uncertainty [33]. As this optimization progresses, the chances of finding a better solution increase. Compared to other techniques such as the grid-search, which is undermined by issues of dimensionality or random-search (where each guess is independent of the previous run), the Bayesian optimization algorithm is fast to compute, enabling a thorough optimization of the hyperparameters [33].

**S8. Supplementary Results: Theory-driven results-Multigroup-structural equation model**:

The multigroup-structural equation model assessed the prediction of cognition and functional capacity based on interactions between combined observable measures of demographic factors, SDH, and CMF (Figure 1B). The Multigroup-structural equation models were run in a subsample of N=15,577 individuals who had completed values in all the SDH, CMF, and demographic variables. The model fitted the data well with acceptable goodness-of-fit indexes (robust CFI= 0.907 and a close fit robust RMSEA= 0.027[0.026 - 0.028]). The standardized scores (a score between 0 and 1 revealing the strength of association) and P values of path regressors between each latent predictor variable and outcome variable are provided below. We also reported the independent path regressors and p-values for Males (M) and Females (F). Results for both cognition and functionality did not reveal significant differences between sex groups as shown by the independent path scores and p-values reported by males and females.

Cognition model: The model showed that cognition was positively predicted by years of education (M:.27, P< .01; F:.28, P< .01) and Social Participation-SDH in females (M:.02, P< .05; F:.10, P< .05). Furthermore, we observed negative association between cognition and a group of social factors including the global SDH factor (M: -.49, P< .001; F:-.54, P< .001) and Socio-economic resources-SDH (M:-.06, P= .098; F:-.17, P< .01). The Social Adversities-SDH and CMF factors did not show a significant effect on cognition. Finally, the global SDH factor was significantly associated with the CMF factor (M:.35, P < .001; F:.25, P< .001, see Supplementary Figure 1).

Functional capacity model: The model showed that a worse global SDH factor (M:-.42, P< .001; F:-.39, P< .001), high-risk CMF (M:-.23, P< .001; F:-.44, P< .001), older age (M:-.22, P< .001; F:-.50, P< .001), more Social Adversities-SDH (M:-.11, P< .05; F:-.08, P< .05) and reduced Socio-economic resources-SDH (M:-.28, P< .01; F:-.11, P< .05) predicted worse functional capacity. Moreover, high scores of Social participation-SDH (M:.10, P<.01; F:-.11, P< .01) explained higher functionality scores (see Supplementary Figure 2).

**Interactions between SDH and CMF**

Our model also revealed complex interactions between demographic, SDH, and CMF in predicting cognition and functional capacity (Table 1). A positive association was found between cognition and functionality (disability for daily life activities and functional mobility; M: 0.24, P < .01; F: 0.19, P < .01). Moreover, we observed reciprocal positive associations between SDH and CMF as individuals with more negative global SDH factor exhibited high-risk CMF (SDH ⇒ CMF M:0.51, P < 0.001; F: 0.36, P < .001; CMF⇒ SDH M: 0.39, P < 0.001; F: 0.29, P < .001) (Supplementary Information S9, Figure 2 and Table S2). Furthermore, older individuals exhibited worse global SDH factor (M: .29, P < .001; F: .20, P < .001), low socio-economic resources (M: -.63, P < .0001; F: .52, P < .001), increased social adversities (M: .06, P< .05; F: .08, P < .01), as well as high-risk CMF (M: .13, P < .001; F: .21, P < .001). Years of education were positively associated with cognition (M: .27, P < .001; F: .28, P < .001). Also, individuals with fewer years of education exhibited worse global SDH factor (M: -.42 P < .0001; F: -.42, P < .0001) and high-risk CMF (M: .15, P < .001; F: .06, P < .01) (Figure 2 and Supplementary Information S9). No sex differences were revealed by the independent path scores and p-values reported by males and females.

**Assessing the isolated role of Cardiometabolic risk factor (CMF) in predicting cognition and functional capacity.**

The model showed that higher scores of CMF were associated with lower scores of cognition (M:-.18, F:-.22, P< .01) and functionality (M:-.11, F:-.13, P< .01, see Supplementary Figure 3).

**Table S1**

| **Author** | **Sample** | **Population** | **Type of study** | **Dependent variables to capture cognition and functionality** | **Independent variables** |
| --- | --- | --- | --- | --- | --- |
| Colombia |  |  |  |  |  |
| Marquez et al., 2022[34] | 23,694 older adults (> 65 yrs) | Representative samples from rural and urban areas in Colombia | Colombian National Survey of Aging (SABE 2015) | Low gait speed and subjective memory complaints in individuals without objective cognitive impairment, and without functional dependency | **Sociodemographic variables:** age, sex, and education.  **Chronic conditions:** self-report of hypertension, coronary artery disease, stroke, cancer, diabetes, obesity, and mental disorders. |
| García-Cifuentes et al., 2020[35] | 23,694 older adults (> 65 yrs) | Representative samples from rural and urban areas in Colombia | Colombian National Survey of Aging (SABE 2015) | Cognitive decline | Gait Speed  **Confounding variables:** sociodemographic (age, education), lifestyle factors (smoking, alcohol consumption, polypharmacy), medical comorbidities (stroke, myocardial infarction, hypertension, mental problems, diabetes). |
| García-Cifuentes et al., 2022[36] | 23,694 older adults (> 65 yrs) | Representative samples from rural and urban areas in Colombia | Colombian National Survey of Aging (SABE 2015) | Cognitive functioning | **Specific association:** Gait Speed and hand grip strength.  **Confounding variables:** Sociodemographic (age and gender), functionality measures; comorbidities (high blood pressure, diabetes, myocardial infarction, stroke, arthropathies, and mental diseases), and anthropometrics (body mass index). |
| Guerrero Barragán et al., 2021[37] | 23,694 older adults (> 65 yrs) | Representative samples from rural and urban areas in Colombia | Colombian National Survey of Aging (SABE 2015) | Cognitive decline  Functionality | **Specific association:** Leisure activities. **Confounding variables:** Sociodemographic (age, area of residence, education, literacy) Social factors (marital status, living alone, health insurance, antecedent of forced displacement, skin color, pension, lifetime occupation, salary). |
| Peréz-Sousa et al., 2021[38] | 23,694 older adults (> 65 yrs) | Representative samples from rural and urban areas in Colombia | Colombian National Survey of Aging (SABE 2015) | Cognitive function | **Specific association:** Handgrip strength **Confounding variables:** Sociodemographic (age and gender), functionality measures, comorbidities (high blood pressure, diabetes, myocardial infarction, stroke, arthropathies, and mental diseases), and anthropometrics (body mass index). |
| O’Donovan et al 2020[39] | 23,694 older adults (> 65 yrs) | Representative samples from rural and urban areas in Colombia | Colombian National Survey of Aging (SABE 2015) | Cognitive function | **Specific association:** Education at childhood. **Confounding variables:** Sociodemographic (age and gender), functionality measures, comorbidities (high blood pressure, diabetes, myocardial infarction, stroke, arthropathies, and mental diseases), and anthropometrics (body mass index). |
| Morros et al., 2017[40] | 23,694 older adults (> 65 yrs) | Representative samples from rural and urban areas in Colombia | Colombian National Survey of Aging (SABE 2015) | Cognitive function | **Specific association:** Diabetes  **Confounding variables:** Sociodemographic (age and gender), functionality measures, comorbidities (high blood pressure, diabetes, myocardial infarction, stroke, arthropathies, and mental diseases), and anthropometrics (body mass index). |
| Borda et al., 2021 | 23,694 older adults (> 65 yrs) | Representative samples from rural and urban areas in Colombia | Colombian National Survey of Aging (SABE 2015) | Cognitive function  Daily life functionality | **Specific association:** Body Mass Index  **Confounding variables:** Sociodemographic (age and gender), functionality measures (Barthel and Lawton scores), comorbidities (high blood pressure, diabetes, myocardial infarction, stroke, arthropathies, and mental diseases), and anthropometrics (body mass index). |
| Studies in LMICs |  |  |  |  |  |
| Mukadam et al., 2019[41] | 16851 older adults (> 65 yrs) | India, China, and six Latin America countries (Cuba, Dominican Republic, Mexico, Peru, Puerto Rico, and Venezuela) | 10/66 Dementia Research surveys | Population attributable fractions (PAFs) of dementia | **Sociodemographic factors:** sex, education, age  **Medical factors:** Hypertension, obesity, hearing loss, diabetes, **Lifestyle factors:** smoking, depression, physical activity  **Social factors:** social contact. |
| Sosa et al 2012[42] | 15376 older adults (> 65 yrs) | India, China, and six Latin America countries (Cuba, Dominican Republic, Mexico, Peru, Puerto Rico, and Venezuela) | 10/66 Dementia Research surveys | Mild cognitive impairment | **Sociodemographic factors:** sex, education, age  **Medical factors:** Hypertension, obesity, hearing loss, diabetes, **Lifestyle factors:** smoking, depression, physical activity  **Social factors:** social contact. |
| Kobayashi et al., 2019[43] | 5059 older adults (> 65 yrs) | South African Rural population | Health and Aging in Africa: A Longitudinal Study of an INDEPTH Community in South Africa | US HRS brief screening instrument for dementia | **Demographic:** age, sex, education.  **Social determinants of health:** marital status, employment, house conditions.  **Medical factors:** self-reported cardiometabolic factors  **Lifestyle factors:** alcohol consumption. |
| Kim et al., 2021[44] | 9836 older adults (> 65 yrs) | Korean urban population | National Survey of Older Koreans (NSOK) | Adapted Cognitive screening | **Demographic:** age, sex, education.  **Social determinants of health:** marital status, employment, house conditions. Rural or urban environment.  **Medical factors:** self-reported cardiometabolic factors  **Lifestyle factors:** alcohol consumption. |
| Miu et al., 2016[45] | 2315 older adults (> 65 yrs) | Mexican urban and rural population | World Health Organization's Study on global AGEing and adult health (WHO SAGE) | Adapted Cognitive screening | **Demographic:** age, sex, education.  **Social determinants of health:** marital status, employment, house conditions. Rural or urban environment  **Medical factors:** self-reported cardiometabolic factors  **Lifestyle factors:** alcohol consumption. |
| Larnyo et al., 2022[46] | 12,430 older adults (> 65 yrs) | China, Ghana, India, Russian Federation, and South Africa. | Global AGEing and Adult Health (SAGE) | Adapted Cognitive screening | **Demographic:** age, sex, education.  **Social determinants of health:** marital status, employment, house conditions.  **Medical factors:** self-reported cardiometabolic factors  **Lifestyle factors:** alcohol consumption, smoking, diet habits. |
| Fernandez-Niño etal., 2018[47] | 16,220 older adults (> 60 yrs) | China, Ghana, India, Russian Federation, and South Africa. | World Health Organization [WHO] Study on Global Ageing and Adult Health | Mental health status | **Demographic:** age, sex, education.  **Social determinants of health:** marital status, employment, house conditions.  **Medical factors:** self-reported cardiometabolic factors  **Lifestyle factors:** alcohol consumption, smoking, diet habits. |
| Lestari et al., 2019[48] | 36,428 older adults (> 65 yrs) | China, Ghana, India, Russian Federation, and South Africa. | World Health Organization (WHO) Study on global AGEing and adult health Wave 1 (2007–2010) | Daily life basic activities | **Demographic:** age, sex, education.  **Social determinants of health:** marital status, employment, house conditions.  **Medical factors:** self-reported cardiometabolic factors  **Lifestyle factors:** alcohol consumption, smoking, diet habits. |
| Zhang et al., 2016[49] | 5362 older adults (> 65 yrs) | Provinces from China | Chinese longitudinal Healthy Longevity Survey (CLHLS), | Composed cognitive screening | **Demographic factors:** Sex, age, marital status, economic situation, area.  **Social determinants of health:** living arrangement, participation in social activities  **Lifestyle factors:** Smoking, drinking, doing housework, physical exercise. **Chronic diseases:** hypertension, arthritis, stroke, digestive system diseases. **Psychiatric problems:** Fearful or anxious, lonely and isolated |

**Table S2.** Latent and observed variables used in the multigroup structural equation model

| Latent variables | Observed  variables | Estimate | Standard  error | P-value | Standardized  Estimate  (Factor loadings) |
| --- | --- | --- | --- | --- | --- |
| Cognition | Orientation | .249 | .011 | .000 | M: .439 / F: .402 |
|  | Counting | .289 | .014 | .000 | M: .267 / F: .275 |
|  | Language | .460 | .018 | .000 | M: .567 / F: .623 |
|  | Recall | .068 | .006 | .000 | M: .109 / F: .134 |
|  |  |  |  |  |  |
| Functionality measures | Lawton | .607 | .030 | .000 | M: .254 / F: .319 |
|  | Barthel | 1.058 | .042 | .000 | M: .451 / F: .404 |
|  | Gait speed | .096 | .004 | .000 | M: .503 / F: .621 |
| SDH | Social resources | .596 | .019 | .000 | M: .683 / F: .639 |
|  | Floor housing | .712 | .023 | .000 | M: .800 / F: .773 |
|  | Housing resources | .114 | .017 | .000 | M: .136 / F: .151 |
|  | Maltreatment | .070 | .018 | .000 | M: .083 / F: .074 |
|  | Discrimination | .038 | .029 | .000 | M: .045 / F: .044 |
|  | Isolation | .076 | .018 | .000 | M: .091 / F: .082 |
|  | Groups participation | .032 | .014 | .000 | M: .038 / F: .031 |
|  | Social help | -.435 | .020 | .000 | M: -.502 / F: -.443 |
|  | Voluntary services | .039 | .014 | .000 | M: .046 / F: .054 |
| SDH-Socio-economic resource | Social resources | .304 | .027 | .000 | M: .377 / F: .335 |
|  | Floor housing | .442 | .040 | .000 | M: .538 / F: .493 |
|  | Housing resources | .020 | .013 | .124 | M: .025 / F: .027 |
| SDH-Social Adversities | Maltreatment | .737 | .035 | .000 | M: .735 / F: .687 |
|  | Discrimination | .826 | .036 | .194 | M: .825 / F: .837 |
|  | Isolation | .719 | .034 | .000 | M: .717 / F: .676 |
| SDH-Social Participation | Groups participation | .786 | .057 | .027 | M: .786 / F: .683 |
|  | Social help | .206 | .021 | .000 | M: .200 / F: .184 |
|  | Voluntary services | .641 | .047 | .000 | M: .642 / F: .774 |
| CMF | Diabetes | .391 | .028 | .000 | M: .399 / F: .286 |
|  | Body Mass Index | 2.226 | .124 | .000 | M: .563 / F: .491 |
|  | Cardiovascular risk | .006 | .002 | .009 | M: .035 / F: .037 |

**Table S3**

|  | | | |  |  |
| --- | --- | --- | --- | --- | --- |
| Features | Estimate | t value | Level of significance | | p value |
| Intercept | 18.745898 | 74.79435337 | **** | | 0.0000000 |
| Mental Problems | 0.798187 | 15.28702915 | **** | | 0.0000000 |
| Live Alone | 0.551534 | 10.68739608 | **** | | 0.0000000 |
| Physical activity | -0.04905 | -1.579015698 | ns | | 0.1143464 |
| Education | 0.306049 | 7.863347946 | **** | | 0.0000000 |
| Falls | 0.167426 | 5.1642076 | **** | | 0.0000002 |
| House Condition | 0.141083 | 21.62882394 | **** | | 0.0000000 |
| Age | -0.138734 | -377.7378795 | **** | | 0.0000000 |
| Alcohol consumption | 0.135632 | 3.767028181 | *** | | 0.0001656 |
| Heart Disease | -0.073843 | -1.703148433 | ns | | 0.0885540 |
| Smoking status | -0.377009 | -9.690687922 | **** | | 0.0000000 |
| Sex | 0.024346 | 0.7362665561 | ns | | 0.4615761 |
| Hypertension | -0.012085 | 0.3854114164 | ns | | 0.6999362 |
| Diabetes | 0.009045 | 0.2229883251 | ns | | 0.8235466 |

**Table S4**

|  | | | |  |  |
| --- | --- | --- | --- | --- | --- |
| Features | Estimate | t value | Level of significance | | p value |
| Intercept | 13.194268 | 84.4764 | **** | | 0 |
| Physical activity | -0.065793 | -3.3987 | *** | | 0.0006783189302 |
| Mental Problems | 0.621358 | 19.0962 | **** | | 0 |
| Live Alone | 0.389371 | 12.1074 | **** | | 0 |
| Falls | 0.315137 | 15.5979 | **** | | 0 |
| Heart Disease | 0.257111 | 9.516 | **** | | 0 |
| Diabetes | 0.180129 | 7.1261 | **** | | 1.06E-12 |
| Sex | 0.151666 | 7.3601 | **** | | 1.90E-13 |
| Alcohol consumption | 0.118496 | 5.2811 | **** | | 1.30E-07 |
| Hypertension | 0.094222 | 4.8217 | **** | | 1.43E-06 |
| Age | -0.09101 | -397.6349 | **** | | 0 |
| Smoking status | -1.188799 | -49.0342 | **** | | 0 |
| Education | 0.045237 | 1.8651 | ns | | 0.06218397596 |
| House Condition | 0.029594 | 7.2804 | **** | | 3.44E-13 |

**Supplementary Figures**


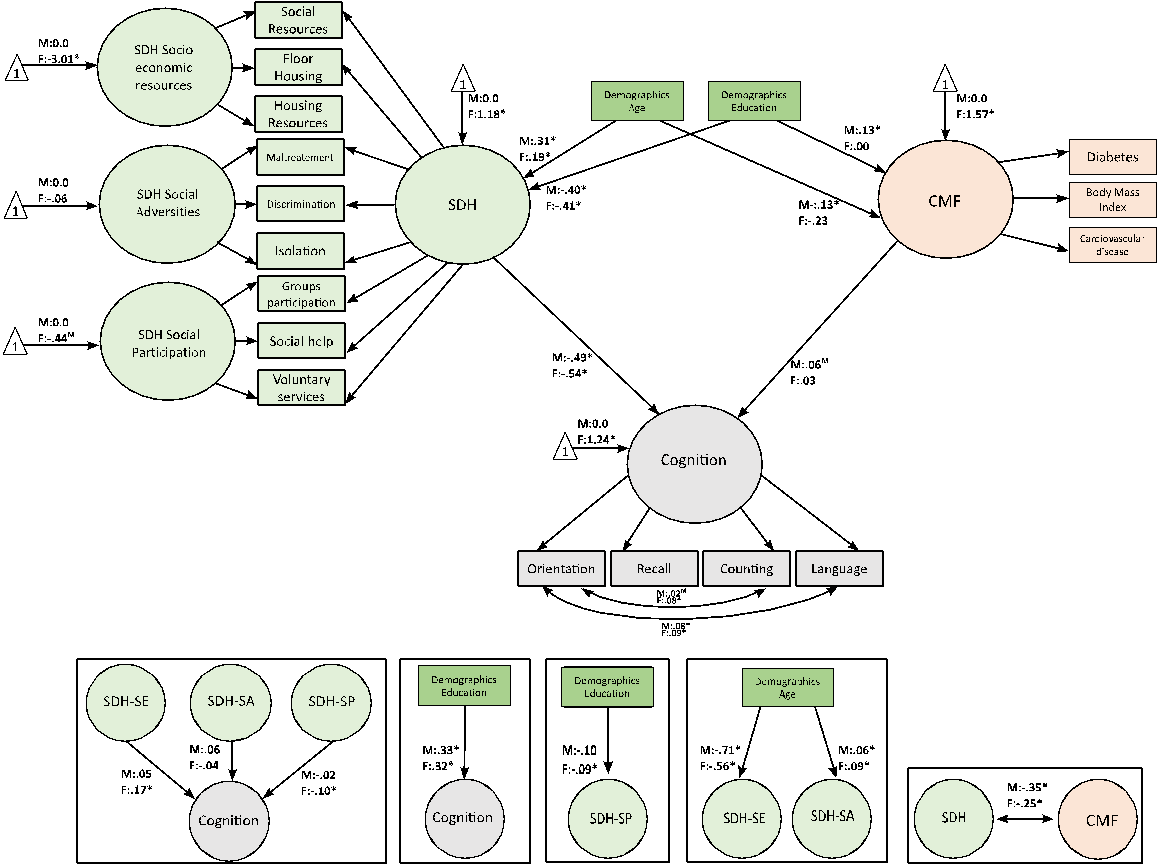


**Supplementary Figure 1:** Structural equation modeling of the impact of inequities on cognition. Panel A shows the SEM of cognition and functionality assessing different SDG, SDH, and CMF predictors. Panel B in the two left images shows the specific predictive values of each of the SDH domains (*SDH-Socio-economic resources*, SDH-S*ocial Adversities* and *SDH-Social Participation*) on cognition and functionality. Panel B in the two right images revealed the prediction values of Age and Years of education on cognition and functionality. For identification of the model, factor means were fixed to zero in males (“M”) and freely estimated for females (“F”). These estimated factor means are expressed as standard deviation units (SD). For example, the factor mean for CMF indicates that the females scored 1.58 SD higher than males (p < 0.001). *: denotes significant effects or sex differences in factor means (p > 0.05). For simplicity, residual variances and observed intercepts are not shown in the figure. SDH: Global Social determinants of health Factor; SDH-SE: Social determinants of health- socio-economic resources; SDH-SA: Social determinants of health- social adversities; SDH-SP: Social determinants of health- social-participation; Education: years of education; CMF: Cardiometabolic Factors.


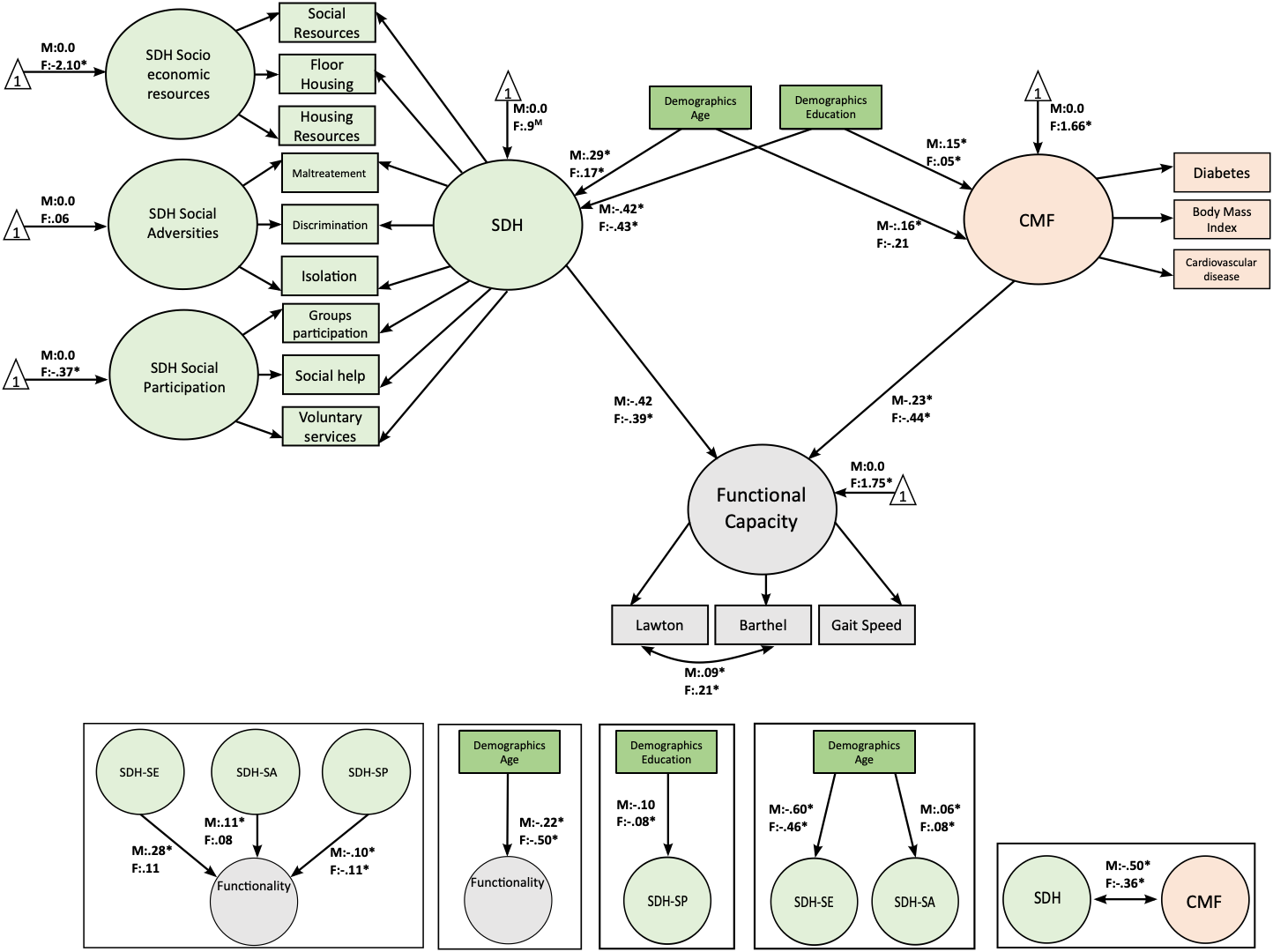


**Supplementary Figure 2**:Structural equation modeling of the impact of inequities on functional capacity. Panel A shows the SEM of cognition and functionality assessing different SDG, SDH, and CMF predictors. Panel B in the two left images shows the specific predictive values of each of the SDH domains (SDH-Socio-economic resources, SDH-Social Adversities and SDH-Social Participation) on cognition and functionality. Panel B in the two right images revealed the prediction values of Age and Years of education on cognition and functionality. For identification of the model, factor means were fixed to zero in males (“M”) and freely estimated for females (“F”). These estimated factor means are expressed as standard deviation units (SD). For example, the factor mean for CMF indicates that the females scored 1.58 SD higher than males (p < 0.001). *: denotes significant effects or sex differences in factor means (p > 0.05). For simplicity, residual variances and observed intercepts are not shown in the figure. SDH: Global Social determinants of health Factor; SDH-SE: Social determinants of health- socio-economic resources; SDH-SA: Social determinants of health- social adversities; SDH-SP: Social determinants of health- social-participation; Education: years of education; CMF: Cardiometabolic Factors.


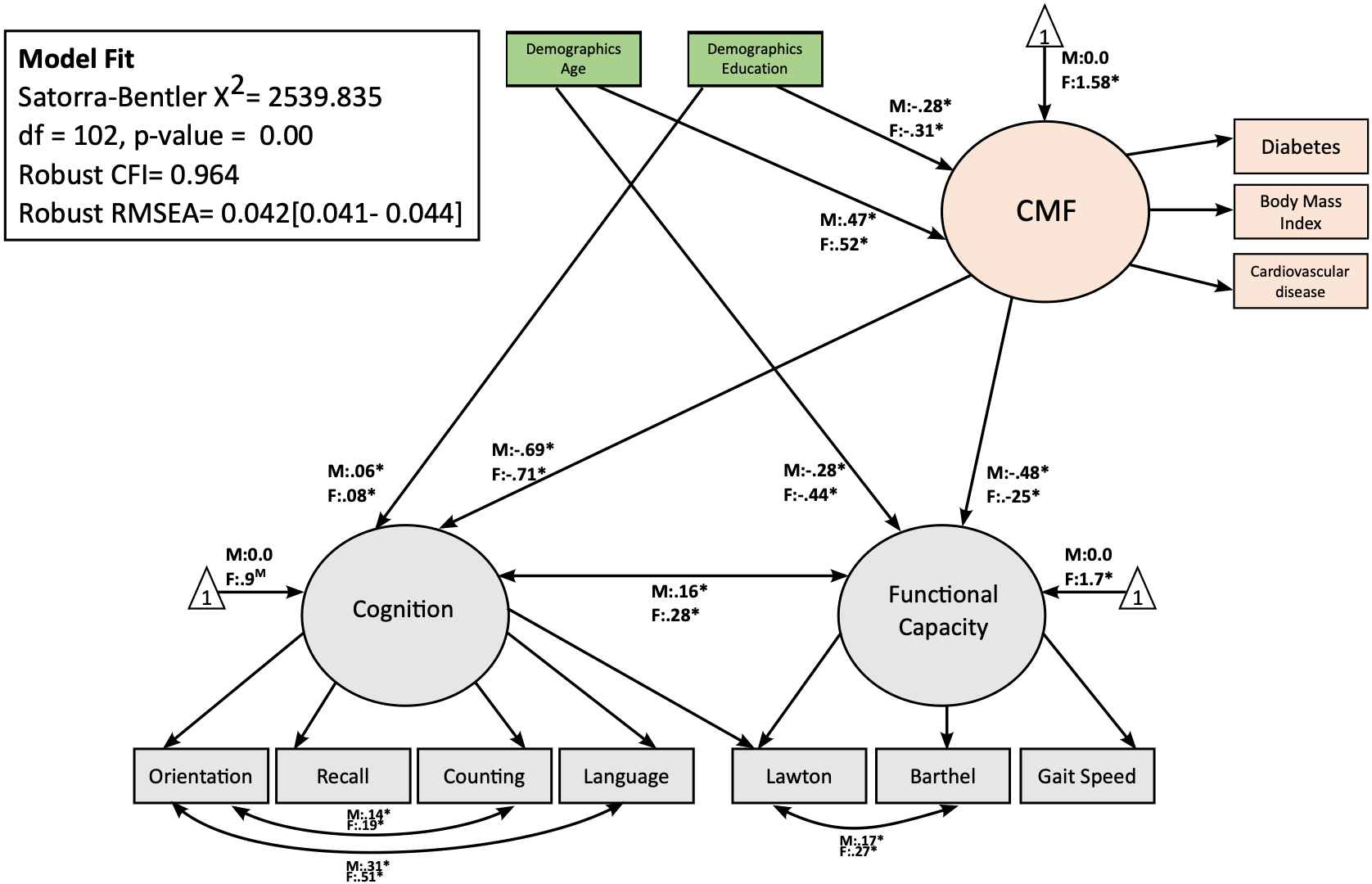


**Supplementary Figure 3**: Structural equation modeling of the impact of cardiometabolic factors on cognition and functional capacity. For identification of the model, factor means were fixed to zero in males (“M”) and freely estimated for females (“F”). These estimated factor means are expressed as standard deviation units (SD). For example, the factor mean for CMF indicates that the females scored 1.58 SD higher than males (p < 0.001). *: denotes significant effects or sex differences in factor means (p > 0.05). CMF: Cardiometabolic Factors.


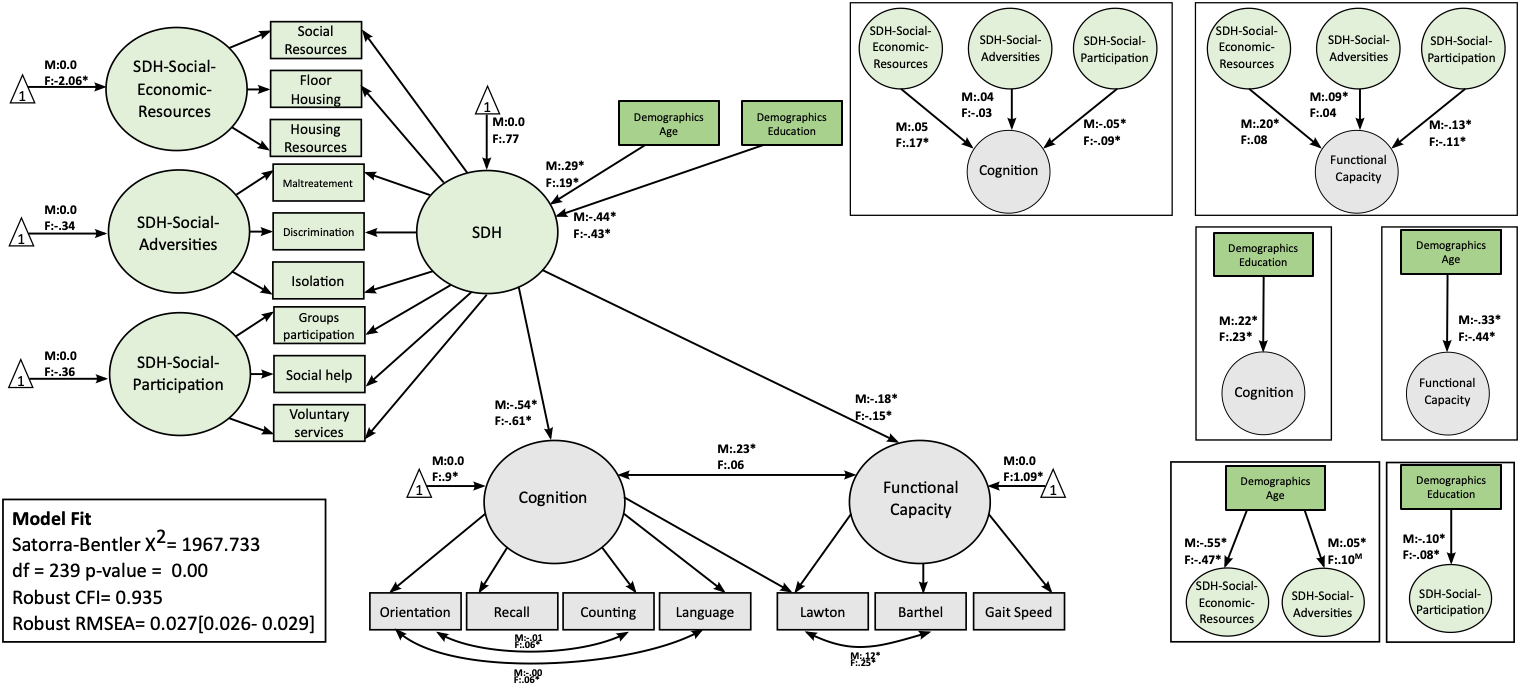


**Supplementary Figure 4:** Structural equation modeling of the impact of social determinants of health on cognition and functional capacity. For identification of the model, factor means were fixed to zero in males (“M”) and freely estimated for females (“F”). These estimated factor means are expressed as standard deviation units (SD). For example, the factor mean for SDH indicates that the females scored 0.77 SD higher than males (p < 0.001). *: denotes significant effects or sex differences in factor means (p > 0.05). SDH: Social determinants of health.

**Supplementary References**

1. Massy-Westropp, N.M., et al., *Hand Grip Strength: age and gender stratified normative data in a population-based study.* BMC Res Notes, 2011. **4**: p. 127.

2. West, S.G., A.B. Taylor, and W. Wu, *Model fit and model selection in structural equation modeling.* Handbook of structural equation modeling, 2012. **1**: p. 209-231.

3. Schermelleh-Engel, K., H. Moosbrugger, and H. M¸ller. *Evaluating the Fit of Structural Equation Models: Tests of Significance and Descriptive Goodness-of-Fit Measures*. 2003.

4. Vandenberg, R.J., *Introduction: Statistical and Methodological Myths and Urban Legends:Where, Pray Tell, Did They Get This Idea?* Organizational Research Methods, 2006. **9**(2): p. 194-201.

5. Little, T., *Model fit, sample size, and power.* Longitudinal SEM: Individual Difference Panel Models, 2013.

6. Browne, M.W. and R. Cudeck, *Alternative ways of assessing model fit.* Sociological methods & research, 1992. **21**(2): p. 230-258.

7. Cohen, J. *Quantitative methods in psychology: A power primer*. in *Psychological bulletin*. 1992. Citeseer.

8. Cheung, G.W. and R.B. Rensvold, *Evaluating goodness-of-fit indexes for testing measurement invariance.* Structural equation modeling, 2002. **9**(2): p. 233-255.

9. Kline, R.B., *Assessing statistical aspects of test fairness with structural equation modelling.* Educational Research and Evaluation, 2013. **19**(2-3): p. 204-222.

10. Team, R.C., *R: A language and environment for statistical computing.* 2013.

11. Epskamp, S., *Reproducibility and replicability in a fast-paced methodological world.* Advances in Methods and Practices in Psychological Science, 2019. **2**(2): p. 145-155.

12. Jorgensen, T., et al., *semTools: Useful tools for structural equation modeling (0.5-3)[Computer software]*. 2020.

13. Rosseel, Y., *Lavaan: An R package for structural equation modeling and more. Version 0.5–12 (BETA).* Journal of statistical software, 2012. **48**(2): p. 1-36.

14. Wickham, H., et al., *Welcome to the Tidyverse.* Journal of open source software, 2019. **4**(43): p. 1686.

15. Hu, L.t. and P.M. Bentler, *Cutoff criteria for fit indexes in covariance structure analysis: Conventional criteria versus new alternatives.* <http://dx.doi.org/10.1080/10705519909540118>, 2009.

16. Shi, D., T. Lee, and A. Maydeu-Olivares, *Understanding the model size effect on SEM fit indices.* Educational and psychological measurement, 2019. **79**(2): p. 310-334.

17. Savalei, V., *On the computation of the RMSEA and CFI from the mean-and-variance corrected test statistic with nonnormal data in SEM.* Multivariate behavioral research, 2018. **53**(3): p. 419-429.

18. Folstein, M.F., S.E. Folstein, and P.R. McHugh, *"Mini-mental state". A practical method for grading the cognitive state of patients for the clinician.* J Psychiatr Res, 1975. **12**(3): p. 189-98.

19. Rojas-Gualdrón, D.F., et al., *Rasch analysis of the Mini Mental State Examination (MMSE) in older adults in Antioquia, Colombia.* CES Psicología, 2017. **10**(2): p. 17-27.

20. Santacruz Escudero, J.M., et al., *Neuropsychiatric Symptoms as Predictors of Clinical Course in Neurodegeneration. A Longitudinal Study.* Front Aging Neurosci, 2019. **11**: p. 176.

21. Yi, Y., et al., *Is Barthel Index Suitable for Assessing Activities of Daily Living in Patients With Dementia?* Frontiers in Psychiatry, 2020. **11**(282).

22. Donnelly-Kehoe, P.A., et al., *Looking for Alzheimer's disease morphometric signatures using machine learning techniques.* Journal of Neuroscience Methods, 2017.

23. Menze, B.H., et al., *A comparison of random forest and its Gini importance with standard chemometric methods for the feature selection and classification of spectral data.* BMC bioinformatics, 2009. **10**(1): p. 1-16.

24. Kaufmann, T., van der Meer, D., Doan, N. T., Schwarz, E., Lund, M. J., Agartz, I., Alnæs, D., Barch, D. M., Baur-Streubel, R., Bertolino, A., Bettella, F., Beyer, M. K., Bøen, E., Borgwardt, S., Brandt, C. L., Buitelaar, J., Celius, E. G., Cervenka, S., Conzelmann, A., Córdova-Palomera, A., … Westlye, L. T. , *Common brain disorders are associated with heritable patterns of apparent aging of the brain.* Nature neuroscience, 2019. **22**(10): p. 1617-1623.

25. Torlay, L., Perrone-Bertolotti, M., Thomas, E., & Baciu, M., *Machine learning-XGBoost analysis of language networks to classify patients with epilepsy.* Brain informatics, 2017. **4**(3): p. 159-169.

26. Behravan, H., et al., *Machine learning identifies interacting genetic variants contributing to breast cancer risk: A case study in Finnish cases and controls.* Sci Rep, 2018. **8**(1): p. 13149.

27. Zheng, H., J. Yuan, and L. Chen, *Short-Term Load Forecasting Using EMD-LSTM Neural Networks with a Xgboost Algorithm for Feature Importance Evaluation.* Energies, 2017. **10**(8): p. 1168.

28. Torlay, L., et al., *Machine learning-XGBoost analysis of language networks to classify patients with epilepsy.* Brain Inform, 2017. **4**(3): p. 159-169.

29. Mason, L.B., J.; Bartlett, P. L.; Frean, Marcus., *Boosting Algorithms as Gradient Descent in Function Space.* Proceedings of the 12th International Conference on Neural Information Processing Systems, 1999.

30. Xuan, P., et al., *Gradient Boosting Decision Tree-Based Method for Predicting Interactions Between Target Genes and Drugs.* Front Genet, 2019. **10**: p. 459.

31. Zeng, X. and G. Luo, *Progressive sampling-based Bayesian optimization for efficient and automatic machine learning model selection.* Health Inf Sci Syst, 2017. **5**(1): p. 2.

32. Feurer M., H.F., *Hyperparameter Optimization. .* Automated Machine Learning. The Springer Series on Challenges in Machine Learning. Springer., 2019.

33. Poldrack, R.A., et al., *Scanning the horizon: towards transparent and reproducible neuroimaging research.* Nat Rev Neurosci, 2017. **18**(2): p. 115-126.

34. Marquez, I., et al., *Motoric Cognitive Risk Syndrome: Prevalence and Cognitive Performance. A cross-sectional study.* The Lancet Regional Health - Americas, 2022. **8**: p. 100162.

35. Garcia-Cifuentes, E., et al., *The Role of Gait Speed in Dementia: A Secondary Analysis from the SABE Colombia Study.* Dement Geriatr Cogn Disord, 2020. **49**(6): p. 565-572.

36. Garcia-Cifuentes, E., et al., *Muscular Function as an Alternative to Identify Cognitive Impairment: A Secondary Analysis From SABE Colombia.* Front Neurol, 2022. **13**: p. 695253.

37. Guerrero Barragán, A., D. Lucumí, and B. Lawlor, *Association of Leisure Activities With Cognitive Impairment and Dementia in Older Adults in Colombia: A SABE-Based Study.* Frontiers in Neurology, 2021. **12**.

38. Pérez-Sousa, M.Á., et al., *Role for Physical Fitness in the Association between Age and Cognitive Function in Older Adults: A Mediation Analysis of the SABE Colombia Study.* International Journal of Environmental Research and Public Health, 2021. **18**(2): p. 751.

39. O'Donovan, G., et al., *Education in early life markedly reduces the probability of cognitive impairment in later life in Colombia.* Sci Rep, 2020. **10**(1): p. 17685.

40. Morros-González, E., et al., *The elderly with diabetes and associated factors. SABE study, Bogotá, Colombia.* Acta Medica Colombiana, 2017. **42**(4): p. 230-236.

41. Mukadam, N., et al., *Population attributable fractions for risk factors for dementia in low-income and middle-income countries: an analysis using cross-sectional survey data.* Lancet Glob Health, 2019. **7**(5): p. e596-e603.

42. Sosa, A.L., et al., *Prevalence, Distribution, and Impact of Mild Cognitive Impairment in Latin America, China, and India: A 10/66 Population-Based Study.* PLOS Medicine, 2012. **9**(2): p. e1001170.

43. Kobayashi, L.C., et al., *Cognitive Function and Impairment in Older, Rural South African Adults: Evidence from "Health and Aging in Africa: A Longitudinal Study of an INDEPTH Community in Rural South Africa".* Neuroepidemiology, 2019. **52**(1-2): p. 32-40.

44. Kim, J. and E. Cha, *Predictors of Cognitive Function in Community-Dwelling Older Adults by Age Group: Based on the 2017 National Survey of Older Korean Adults.* Int J Environ Res Public Health, 2021. **18**(18).

45. Miu, J., et al., *Factors associated with cognitive function in older adults in Mexico.* Glob Health Action, 2016. **9**: p. 30747.

46. Larnyo, E., et al., *Examining the impact of socioeconomic status, demographic characteristics, lifestyle and other risk factors on adults' cognitive functioning in developing countries: an analysis of five selected WHO SAGE Wave 1 Countries.* International Journal for Equity in Health, 2022. **21**(1): p. 31.

47. Fernández-Niño, J.A., et al., *Work status, retirement, and depression in older adults: An analysis of six countries based on the Study on Global Ageing and Adult Health (SAGE).* SSM Popul Health, 2018. **6**: p. 1-8.

48. Lestari, S.K., et al., *Diversity in the factors associated with ADL-related disability among older people in six middle-income countries: a cross-country comparison.* International journal of environmental research and public health, 2019. **16**(8): p. 1341.

49. Zhang, Y., et al., *Study on Prediction of Activities of Daily Living of the Aged People Based on Longitudinal Data.* Procedia Computer Science, 2016. **91**: p. 470-477.
